# Efficient and Robust Heterostructure CeZrO_x_/NiO‐Ni Inverse Catalyst for Sustainable Photothermal CO_2_ Methanation

**DOI:** 10.1002/advs.202522942

**Published:** 2026-01-30

**Authors:** Chuqiao Song, Zhaohua Wang, Zhouhong Ren, Cheng Yu, Haibo Li, Xin Tang, Houhong Song, Yao Xu, Liangwei Liu, Lili Han, Liwei Chen, Zhifu Qi, Xi Liu, Siyu Yao, Xiao‐nian Li, Xiang Gao, Lili Lin

**Affiliations:** ^1^ State Key Laboratory of Green Chemical Synthesis and Conversion Zhejiang Key Laboratory of Surface and Interface Science and Engineering for Catalysts College of Chemical Engineering Zhejiang University of Technology Hangzhou China; ^2^ Zhejiang Carbon Neutral Innovation Institute & Zhejiang International Cooperation Base for Science and Technology on Carbon Emission Reduction and Monitoring Zhejiang University of Technology Hangzhou China; ^3^ Science and Education Integration College of Energy and Carbon Neutralization Zhejiang University of Technology Hangzhou China; ^4^ Zhejiang Baima Lake Laboratory Co., Ltd. Hangzhou China; ^5^ School of Chemistry and Chemical Engineering In Situ Center for Physical Sciences Frontiers Science Center for Transformative Molecules Shanghai Jiao Tong University Shanghai China; ^6^ State Key Laboratory of Chemical Engineering College of Chemical and Biological Engineering Zhejiang University Hangzhou China; ^7^ Beijing National Laboratory for Molecular Sciences College of Chemistry and Molecular Engineering Beijing China; ^8^ State Key Laboratory of Structural Chemistry Fujian Institute of Research on the Structure of Matter Chinese Academy of Sciences Fuzhou China; ^9^ School of Chemistry and Chemical Engineering Ningxia University Yinchuan China

**Keywords:** CO_2_ methanation, heterostructure engineering, intermittency matching, inverse catalysts, photothermal synergy

## Abstract

Photothermal CO_2_ methanation offers a route to store renewable energy as synthetic methane, yet conventional Ni catalysts typically require intense light or auxiliary heating and show poor tolerance to intermittency. Here, we report a CeZrO_x_/NiO‐Ni inverse catalyst via a heterostructure engineering strategy, featuring a protective NiO interlayer. This tailored architecture achieves 83% single‐pass CO_2_ conversion with >99% CH_4_ selectivity under 0.71 W cm^−2^ irradiation in a continuous‐flow system, without external heating. Notably, it delivers a CH_4_ space‐time yield of 464 mmol·g_cat_^−1^·h^−1^ under natural concentrated sunlight and maintains robust performance over repeated light‐dark cycles, extended air storage, and 100‐g scaled synthesis, demonstrating its potential compatibility with intermittent renewable energy. Mechanistic studies reveal that the sub‐nanometer NiO layer on Ni domains enhances LSPR‐induced heating and hot‐carrier injection while establishing a favorable CeZrO_x_/NiO‐Ni band alignment for charge separation. This heterointerface further promotes CO_2_ activation via *COOH intermediates and electron‐mediated pathways, thereby amplifying photothermal synergy under low‐intensity illumination. This work highlights the critical role of interfacial engineering in advancing solar‐driven energy conversion and provides a catalyst‐level design strategy that could help bridge lab‐scale innovation and future practical applications.

## Graphical abstract

A heterostructured CeZrO_x_/NiO‐Ni inverse catalyst is designed for highly efficient photothermal CO_2_ methanation under low‐intensity light without auxiliary heating. It achieves 464 mmol·g_cat_^−1^·h^−1^ methane space‐time yield under natural concentrated sunlight, maintains stability over light‐dark cycles and extended air storage, and is scalable to 100 g, demonstrating robust adaptability to intermittent renewable energy.

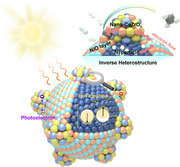

## 1 Introduction

With global methane (CH_4_) demand expected to reach 4–5 trillion cubic meters annually by 2030, converting captured CO_2_ and green hydrogen into synthetic natural gas, i.e. CO_2_ methanation, has attracted growing interest as a potential route for long‐duration energy storage and carbon utilization [1, 2, 3, 4]. Photothermal CO_2_ methanation is a desirable pathway that uses only solar energy as the input, eliminating the need for secondary energy sources [5, 6, 7, 8]. Maximizing solar‐to‐fuel efficiency, however, requires catalysts that couple photothermal heating with photochemical charge transfer, since proton‐coupled CO_2_ reduction can proceed with lower activation barriers when hot carriers are available [9, 10]. Practical deployment further demands scalability and resilience to solar intermittency [11, 12]. Thus, robust catalysts that function under low‐intensity irradiation with inexpensive materials are a key target for solar‐driven methanation [13, 14].

Nickel‐based catalysts are widely employed in thermal methanation due to their low cost and scalability [6, 15, 16], yet their low free‐carrier density and intrinsically lossy plasmon yield weak LSPR [17] compared to plasmonic metals like Au [7], Ag [18], or Cu [19], making Ni‐based photothermal systems reliant on very high light fluxes or external heating (>2 W cm^−2^, ∼20 suns) to reach thermal‐catalysis temperatures (i.e. >300°C) [20, 21, 22]. Structural tactics such as higher Ni loading [23], thermal barrier coatings (e.g., SiO_2_ [6], C [24], or cermets [22]), support modification [25] or blending photothermal additives [8, 26], have aimed to minimize radiative losses and enhance light‐to‐heat efficiency. These routes, while effective thermally, intensify stability demands and shorten hot‐carrier lifetimes,^7^ thereby suppressing low‐barrier, electron‐mediated CO_2_ activation pathways (e.g., CO_2_^·−^ formation [9, 27, 28, 29, 30]). The central challenge is therefore to amplify photothermal effect and promote carrier selectivity at low light intensity, without sacrificing durability or cost. An “inverse” structure, wherein oxide nanoparticles are dispersed on a metallic substrate rather than the conventional dispersion of metal nanoparticles on an oxide support, offers a path to this balance by providing enhanced interfacial density and electronic tunability [31, 32]. In prior work, nano‐oxides dispersed on metallic Ni supports approached theoretical methanation rates near 200°C [31, 33, 34], suggesting that a conductive Ni network can provide efficient heat spreading while the surface oxides mediate CO_2_ activation. This makes it a highly promising candidate that can be further engineered, through targeted strategies tailored for light‐driven reactions, into an efficient photothermal CO_2_ methanation catalyst operable under low‐intensity irradiation (Figure S1a,b). In addition, the high reactivity of metallic Ni compromises stability and air‐storage during irradiation gaps, necessitating designs that tolerate intermittency and enable reliable restart.

Herein, we report a heterostructured CeZrO_x_/NiO‐Ni inverse catalyst that achieves exceptional photothermal CO_2_ methanation under low‐intensity irradiation (Figure S1c), where optimized photothermal synergy enables high performance at low light‐induced temperatures. In a continuous‐flow reactor, the catalyst delivers 83% single‐pass CO_2_ conversion with >99% CH_4_ selectivity under 0.71 W cm^−2^ Xe light without auxiliary heating, approximately 30‐fold higher than that of a conventional Ni/CeZrO_x_ catalyst. Under actual concentrated sunlight, it attains a CH_4_ STY of 464 mmol g_cat_^−1^ h^−1^ with ∼80% conversion and >99% selectivity. The material maintains stable performance during light‐dark cycling, exhibits superior air‐storage durability, ensured by the protective NiO interlayer, and is scalable to 100‐gram batches. These combined attributes underscore the potential of this catalyst design for solar‐driven CO_2_ conversion. Mechanistically, the sub‐nanometer NiO layer on Ni substrate couples plasmonic heating with favorable carrier dynamics, enabling electron‐mediated CO_2_ activation at the CeZrO_x_/NiO‐Ni junction. This rational interface engineering synergistically enhances photothermal synergy, structural stability, and operational adaptability, thereby providing an effective catalyst design strategy for advancing solar‐driven CO_2_ conversion.

## 2 Results and Discussion

### 2.1 Structural Characterization

**FIGURE 1:**
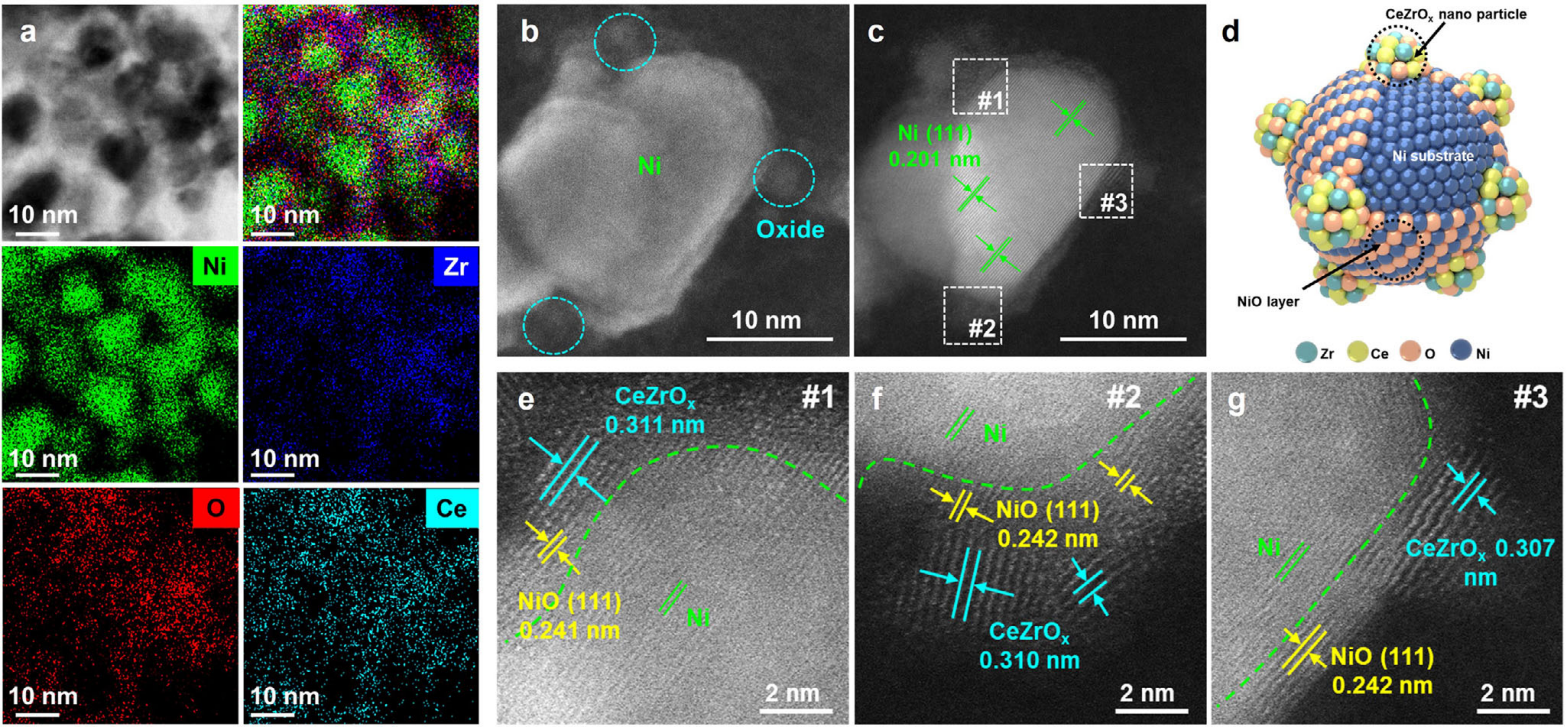
STEM imaging of CeZrO_x_/NiO‐Ni inverse catalyst. (a) STEM image and the corresponding EDS elemental mappings (green: Ni, dark blue: Zr, red: O, light blue: Ce), (b) HAADF‐STEM‐SE image, (c) HAADF‐STEM‐ADF image, (d) schematic diagram, (e‐g) the amplified regions correspond to the #1‐3 region in (c).

CeZrO_x_/NiO‐Ni inverse catalysts were synthesized using a pH‐adjusted oxalate coprecipitation, followed by H_2_ reduction and subsequent 0.5% O_2_ passivation treatment (see Methods, Figures S2, S3 and Table S1). To examine the morphology of the CeZrO_x_/NiO‐Ni inverse catalyst, scanning transmission electron microscopy (STEM) and energy dispersive X‐ray spectroscopy (EDS) were employed. STEM imaging revealed a uniform dispersion of oxide species over the ∼10 nm Ni substrate (Figure 1a), while elemental mapping confirmed the co‐localization of Ce and Zr surrounding the Ni particles. Secondary electron imaging (Figure 1b) and atomic‐resolution annular dark‐field STEM (Figure 1c,e‐g) further illustrated that the Ni particles were encapsulated by a 0.5‐1.0 nm NiO layer. Lattice fringes with a spacing of approximately 0.311 nm were identified, corresponding to the (101) plane of CeZrO_x_ (PDF#38‐1436), confirming the formation of a mixed oxide phase (Figure 1e‐g). These results collectively verify a composite inverse structure in which CeZrO_x_ nanoparticles are anchored onto the Ni substrate via a protective interfacial NiO interlayer (Figure 1d).

**FIGURE 2:**
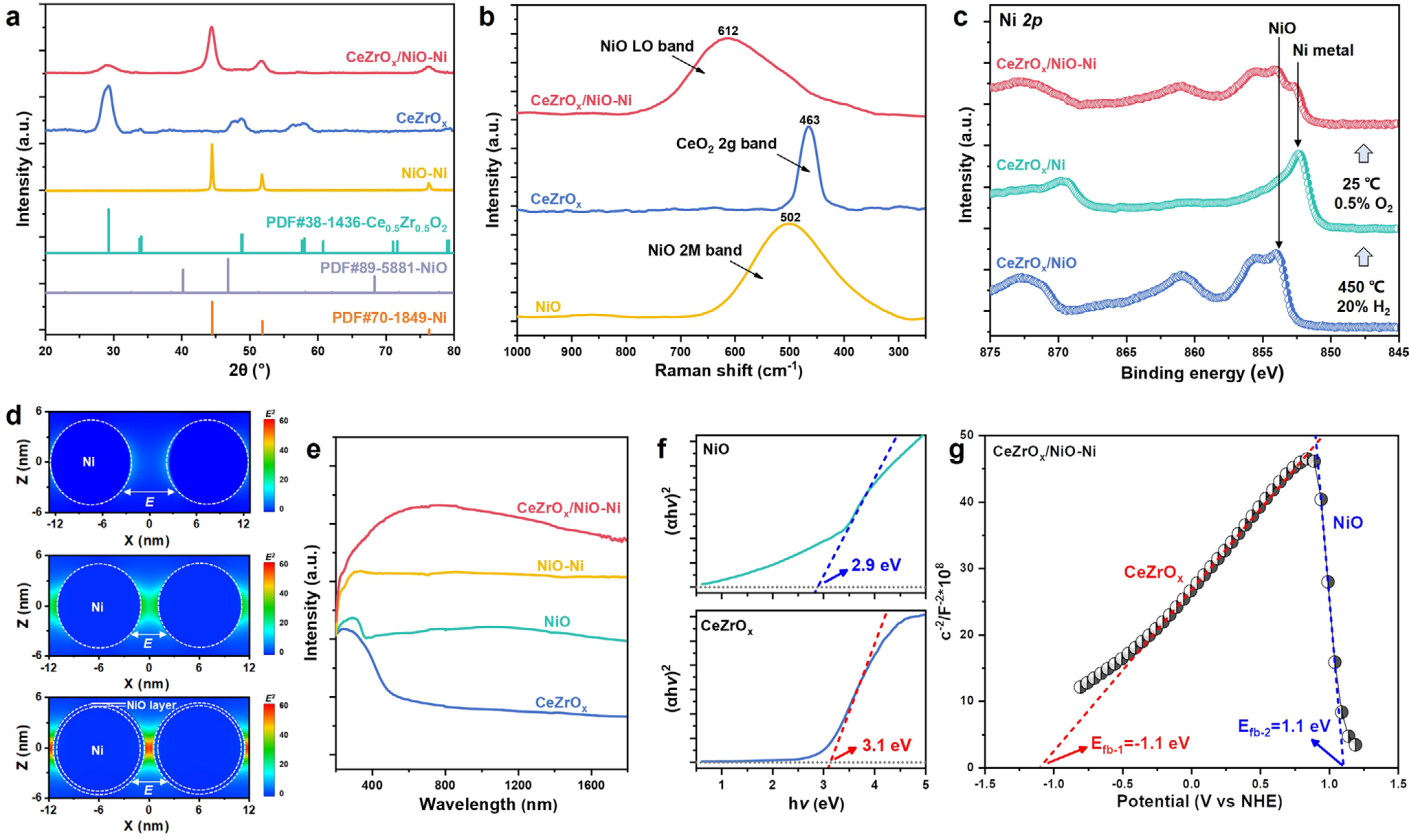
Characterizations of CeZrO_x_/NiO‐Ni inverse catalyst. (a) XRD patterns of the CeZrO_x_/NiO‐Ni, CeZrO_x_ and NiO‐Ni catalysts; (b) Raman spectra of the CeZrO_x_/NiO‐Ni, CeZrO_x_ and NiO catalysts. (c) quasi in situ Ni *2p* XPS spectra of the CeZrO_x_/NiO‐Ni catalyst during the reduction and passivation process; (d) Spatial distribution of the LSPR‐induced enhancement of electric field intensity at 450 nm from 3D FDTD simulation of 10 nm exposed Ni nanoparticles with 4 nm apart (top), 10 nm exposed Ni nanoparticles with 2 nm apart (middle) and 10 nm Ni nanoparticles overcoated by 0.5 nm NiO layer with 2 nm apart (bottom). (e) UV‐vis diffuse reflectance results of the CeZrO_x_/NiO‐Ni, NiO‐Ni, CeZrO_x_ and NiO catalysts. (f) Tauc plots of CeZrO_x_ and NiO catalyst. (g) Mott‐Schottky plot ofCeZrO_x_/NiO‐Ni catalyst.

The structural evolution of the CeZrO_x_/Ni‐Ni catalyst throughout reduction and passivation was systematically investigated. X‐ray powder diffraction (XRD) profile of CeZrO_x_/NiO‐Ni showed the pattern of metallic Ni, together with a typical CeZrO_x_ signature at low 2‐theta angle of 29.3° (Figure 2a). Scherrer equation determined ∼12 nm Ni particle size and more dispersed CeZrO_x_ with ∼5 nm particle size. The absence of distinct NiO reflections suggested the presence of an ultrathin NiO layer after passivation. Compared to the NiO‐Ni reference, CeZrO_x_/NiO‐Ni exhibited broader and less intense Ni peaks, indicative of smaller Ni domains and improved dispersion, likely due to strong metal‐oxide interactions that suppress Ni aggregation. Raman spectroscopy provided further insight into the catalyst structure (Figure 2b). It showed that CeZrO_x_ exhibited the CeO_2_ 2 g band at 464 cm^−1^, while NiO displayed a prominent 502 cm^−1^ two‐magnon (2 M) feature [35, 36]. In CeZrO_x_/NiO–Ni, a dominant 612 cm^−1^ longitudinal optical (LO) band emerged and the 2 M signal vanished, indicative of a sub‐nanometer/defective NiO layer in which lattice disorder activated LO/TO phonons and disrupted Ni^2+^ superexchange [35, 36]. Quasi in situ XPS results revealed that the passivated CeZrO_x_/NiO‐Ni catalyst contained both Ni^0^ and Ni^2+^ species compared to that of fresh and reduced samples (Figure 2c), with metallic Ni accounting for approximately 9.5% of the nickel species (Figure S4 and Table S2), indicating the presence of NiO thin layer over metallic Ni.

To probe the light response of CeZrO_x_/NiO‐Ni, 3D finite‐difference time‐domain (FDTD) simulations were conducted (Figure 2d). Under 450 nm illumination [37], it showed that two 10 nm Ni particles with a 2 nm gap (middle) produce much higher local fields (|E|^2^) than particles 4 nm apart (**top**), indicating stronger LSPR heating in continuous Ni domains [38]. Adding a 0.5 nm NiO layer further raised the peak enhancement from ∼32 to ∼57 (**bottom**). Electron microscopy revealed continuous NiO‐Ni phase in the inverse catalyst (Figure 1a), unlike the discrete Ni dispersion in conventional systems (Figures S5 and S6), enabling collective plasmonic amplification and consistent with higher surface temperatures under irradiation (Figure S7). Complementary UV‐vis absorption spectroscopy revealed that CeZrO_x_/NiO‐Ni exhibited broader and more intense light absorption across the spectrum compared to individual components, attributed to its inverse structural configuration (Figure 2e) [21]. Based on Tauc plots, the bandgap energies of CeZrO_x_ and NiO were estimated to be 3.1 and 2.9 eV, respectively (Figure 2f). Subsequent Mott‐Schottky analyses (Figure 2g) identified CeZrO_x_ as an n‐type and NiO as a p‐type semiconductor, confirming the formation of a p‐n junction within the CeZrO_x_/NiO‐Ni interface, and the energy band diagram could be constructed based on flat‐band potentials (Figure S8).

### 2.2 Catalytic Performance Evaluation

**FIGURE 3:**
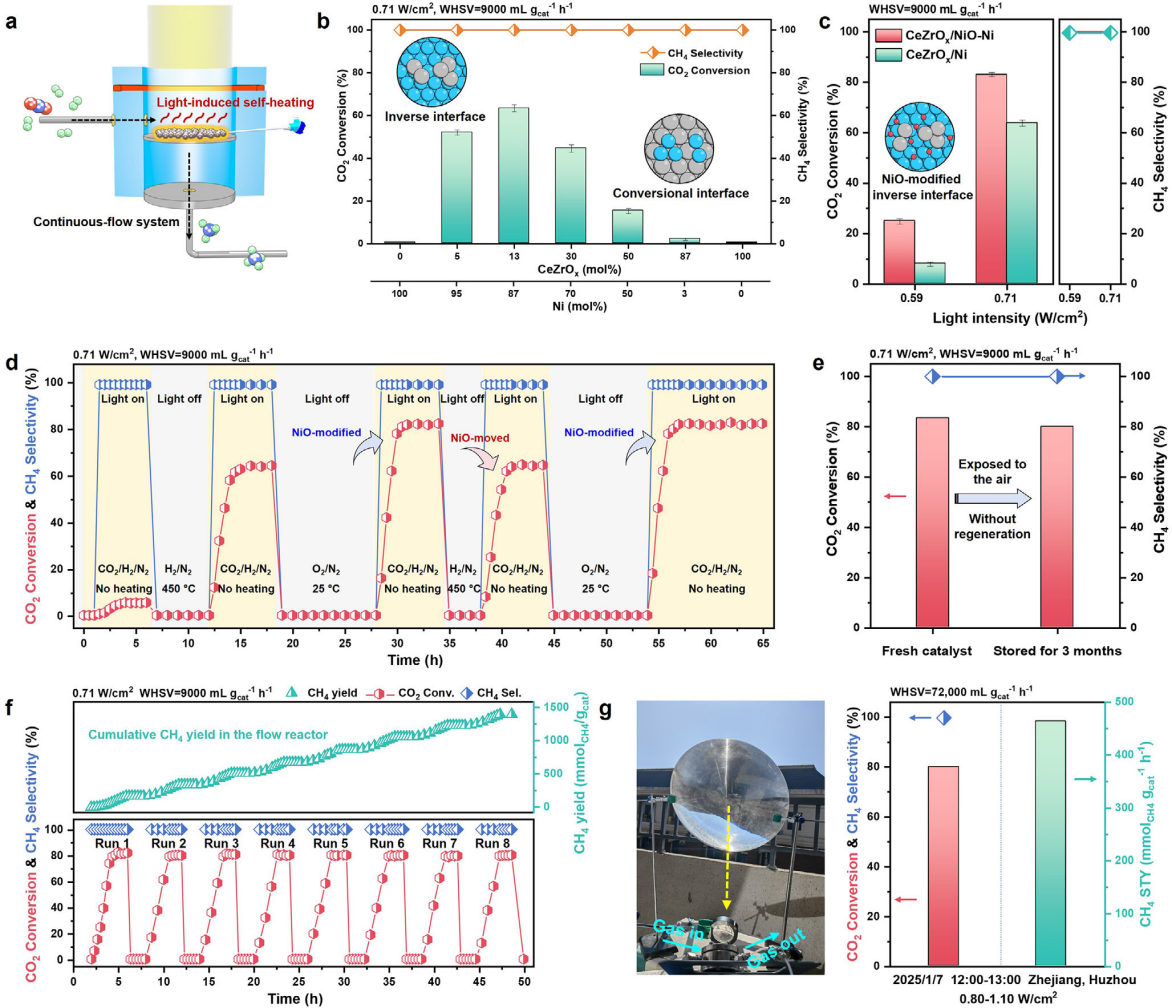
Photothermal catalytic performance of CO_2_ methanation over CeZrO_x_/NiO‐Ni inverse catalyst. (a) Schematic of the photothermal performance evaluation setup; (b) CO_2_ conversion and CH_4_ selectivity on the CeZrO_x_/Ni catalysts with different molar% of CeZrO_x_ at 0.71 W/cm^2^ light intensity, the error bars show the deviation of CO_2_ conversion based on three repeated experiments; (c) CO_2_ conversion and CH_4_ selectivity on the CeZrO_x_/Ni and CeZrO_x_/NiO‐Ni catalysts within light intensities of 0.59 and 0.71 W/cm^2^; (d) CO_2_ conversion and CH_4_ selectivity on different states of CeZrO_x_/NiO‐Ni catalyst by in situ continuous reduction and passivation; (e) CO_2_ conversion and the CH_4_ selectivity of the catalyst after long‐term storage in air and reuse without regeneration. Reaction conditions for the catalytic test of b‐e: WHSV = 9000 mL g_cat_^−1^ h^−1^, 300 W Xe lamp (λ = 320–1000 nm), light intensity: 0.59/0.71 W/cm^2^, no external heating, CO_2_:H_2_:N_2_ = 18:72:10, total flow rate = 12 mL/min, p = 0.1 MPa). (f) Catalyst stability in terms of the CO_2_ conversion, CH_4_ selectivity and CH_4_ cumulative yield for 50 h and 8 times of light on‐off cycle. (g) Photothermal process under outdoor sunlight irradiation. This test was operated in Huzhou, Zhejiang, China at 12:00–13:00, seventh January, 2025, sunlight intensity was 0.8–1.1 W/cm^2^, the WHSV = 72 000 mL g_cat_^−1^ h^−1^.

Photothermal CO_2_ methanation was evaluated in a custom continuous‐flow reactor at ambient pressure and a weight hourly space velocity (WHSV) of 9000 mL g_cat_^−1^ h^−1^ (Figure 3a; Figure S9). The reactor was illuminated by a 300 W Xe lamp (320‐1000 nm, Figure S10) without external heating. Unless noted otherwise, *x* mol% denotes the loading of the corresponding component. Systematic variation of CeZrO_x_ loading in the reduced CeZrO_x_/Ni catalysts revealed a volcano‐type dependence of CO_2_ conversion, with a maximum of 65% conversion achieved at 13 mol% CeZrO_x_, in sharp contrast to the mere 2% conversion observed for conventional 87 mol% CeZrO_x_ with 13 mol% Ni (Figure 3b). With the optimized oxidation treatment to form a NiO layer (exposure to 0.5% O_2_ at 25°C for 8 h, Figure S11), the CeZrO_x_/Ni‐NiO exhibited a CO_2_ conversion of 25% at a low light intensity of 0.59 W cm^−2^ (the light‐induced catalyst‐bed temperature was ∼160°C, Figure S7), which was threefold higher than that of CeZrO_x_/Ni under identical irradiation conditions (Figure 3c). When the light intensity was increased to 0.71 W cm^−2^ (the catalyst‐bed temperature reached ∼200°C), CO_2_ conversion on the CeZrO_x_/NiO‐Ni further elevated to 83% exceeding that of 64% on the CeZrO_x_/Ni, comparable to thermocatalytic inverse catalysts at the same temperature [31], highlighting the crucial role of the NiO layer in enhancing photothermal catalytic performance. The NiO‐modified catalyst also demonstrated the advantages of the inverse structure, as the CO_2_ conversion over CeZrO_x_/NiO‐Ni increased to 90% when the light intensity increased to 1.06 W cm^−2^, compared to merely 12% for conventional NiO‐Ni/CeZrO_x_ (Figure S11c). At an elevated WHSV of 288,000 mL•g_cat_^−1^ h^−1^, the CeZrO_x_/NiO‐Ni catalyst achieved a remarkable CH_4_ STY of 1024 mmol_CH4_•g_cat_^−1^ h^−1^ with ∼45% CO_2_ conversion at 0.71 W cm^−2^ irradiation (Figures S12 and S13), outperforming most previously reported non‐noble metal photothermal catalysts and rivalling state‐of‐the‐art noble metal systems.

To prove the significance of NiO layer in promoting CO_2_ conversion under light, sequential in situ reduction and passivation cycles were conducted in a continuous‐flow reactor (Figure 3d). Under illumination at 0.71 W cm^−2^, the calcined CeZrO_x_/NiO catalyst exhibited a CO_2_ conversion of 5%, which sharply increased to 64% following reduction, corresponding to the formation of the CeZrO_x_/Ni inverse structure. Subsequent exposure to 0.5% O_2_ at 25°C for 8 h led to the gradual formation of a NiO passivation layer, resulting in a further increase in CO_2_ conversion to 83% under the same illumination. This performance enhancement was consistently maintained across multiple NiO modified‐moved cycles, confirming the robust promotional role of the NiO layer, where the long‐term operation also confirmed the structural stability of the NiO interlayer. The advantage of NiO modification was further observed in other inverse systems, such as CeO_2_/NiO‐Ni and ZrO_2_/NiO‐Ni (Figure S14), indicating the broad applicability of this strategy for improving photothermal CO_2_ methanation. In addition to the activity promotion, the NiO layer imparted improved air tolerance to the inverse catalyst, significantly reducing its sensitivity to atmospheric oxygen. After three months of air exposure, the CeZrO_x_/NiO‐Ni catalyst retained full activity without regeneration (Figure 3e), demonstrating excellent stability under prolonged idle conditions.

Practical photothermal CO_2_ hydrogenation demands high activity and durable stability under intermittent renewable operation. Accordingly, a 50‐h durability test incorporating eight light on/off cycles was performed on CeZrO_x_/NiO‐Ni, during which the catalyst preserved its initial activity and delivered a cumulative 1,394 mmol_CH4_ g_cat_^−1^. Post‐reaction XRD analysis confirmed the structural integrity of the catalyst (Figure S15), underscoring its resilience under fluctuating conditions. Notably, performance under concentrated outdoor sunlight using Fresnel and plano‐convex lenses (0.80–1.10 W cm^−2^) was assessed, where the inverse catalyst achieved a CO_2_ conversion of ∼80%, CH_4_ selectivity exceeding 99%, and a CH_4_ STY of 464  mmol_CH4_ g_cat_^−1^ h^−1^ (Figure 3g; Figures S16 and S17). In addition, the synthesis of CeZrO_x_/NiO‐Ni was successfully scaled up to the 100‐gram level, with the resulting catalysts retaining activity comparable to laboratory scale (Figure S18). The combination of high photothermal efficiency, robust stability, and scalable fabrication supports the CeZrO_x_/NiO‐Ni catalyst as a promising candidate for further investigation in solar‐driven CO_2_ conversion applications.

### 2.3 Mechanism Studies of Photothermal CO_2_ Hydrogenation

**FIGURE 4:**
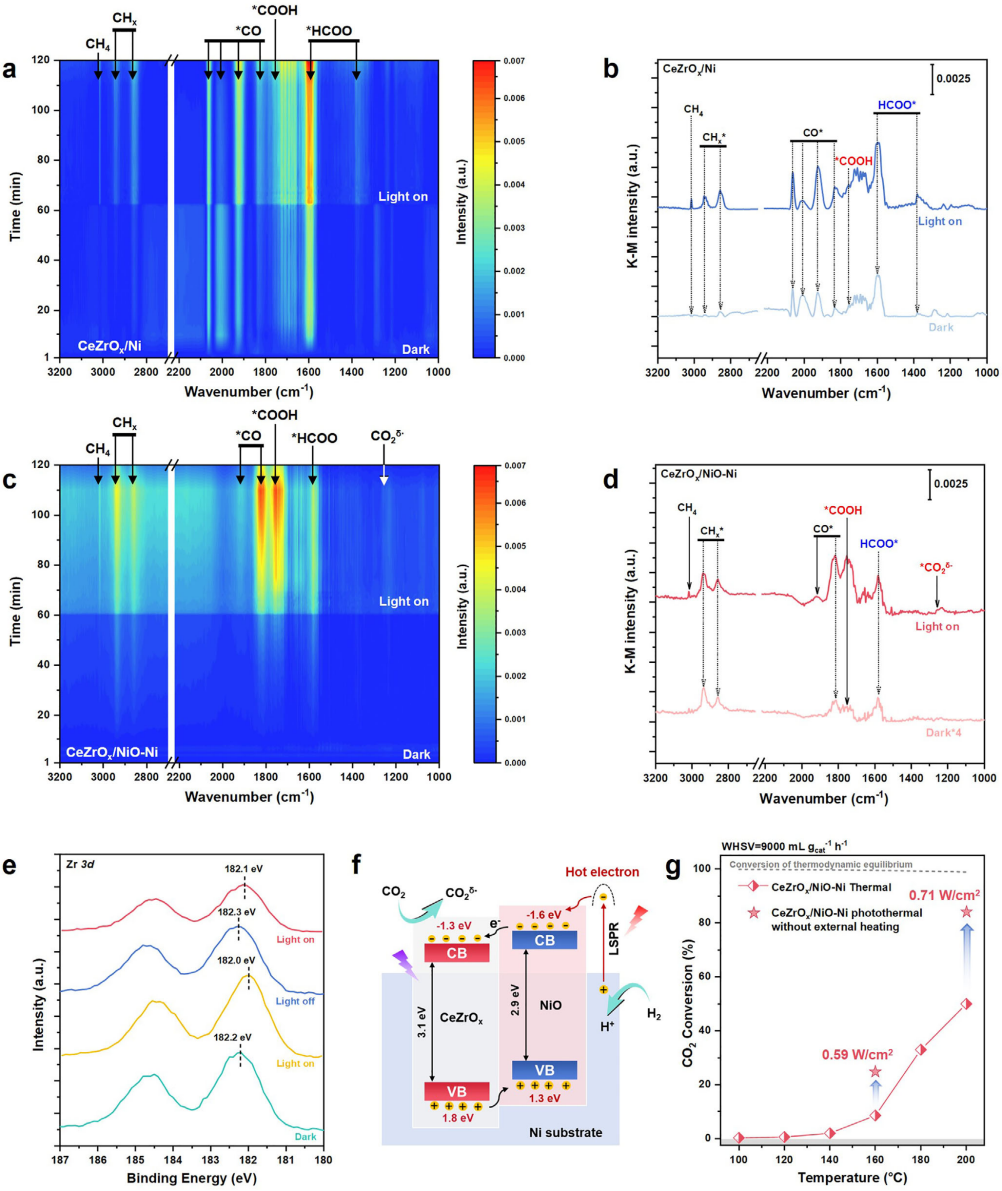
Mechanism studies of CO_2_ photothermal conversion on CeZrO_x_/Ni and CeZrO_x_/NiO‐Ni inverse catalyst. In situ DRIFTS studies on (a) CeZrO_x_/Ni and (c) CeZrO_x_/NiO‐Ni under CO_2_+H_2_ at 150°C, the light was introduced by a fiber after 60 minutes’ reaction, the temperature fluctuation by light could be ignored; DRIFTS spectra of steady state before and after 60 minutes’ irradiation on the (b) CeZrO_x_/Ni and (d) CeZrO_x_/NiO‐Ni catalysts under CO_2_+H_2_; (e) NAP‐XPS results of Zr *3d* on CeZrO_x_/NiO‐Ni with light off‐on switching under Ar atmosphere. (f) Schematic of electrons and holes flow on CeZrO_x_/NiO‐Ni catalyst under irradiation. (g) Comparison of the activity between thermal and photothermal process on the CeZrO_x_/NiO‐Ni inverse catalyst.

In situ DRIFTS on the CeZrO_x_/Ni and CeZrO_x_/NiO‐Ni were used to elucidate photothermal synergistic effects and reaction pathways in photothermal CO_2_ methanation (Figure 4a‐g). A Xe lamp was coupled to the IR reaction cell via optical fiber (Figure S19). To suppress light‐induced heating, the catalyst bed was diluted tenfold with quartz sand and maintained at 150°C. (Figure 4a,b). In the dark, reduced CeZrO_x_/Ni displayed bands assigned to CH_x_ species (2937, 2861 cm^−1^) [39, 40], linear and bridged Ni‐CO^*^ species (2060‐1820) [41], bicarbonates (1661, 1236‐1215 cm^−1^), carbonates (1567, 1280 cm^−1^) [42], relatively weak carboxylate (*COOH, 1758 cm^−1^) and intense formate (HCOO^*^, 1580 and 1380 cm^−1^) species [43, 44]. After reaching steady state (∼60 min), illumination strengthened all intermediate signals and yielded a CH_4_ feature at 3017 cm^−1^ (Figure 4a,b), indicating that light‐enhanced methanation proceeds along a pathway broadly consistent with thermal operation [44, 45]. In contrast, CeZrO_x_/NiO‐Ni exhibited a more pronounced light‐dark difference (Figure 4c,d). In the dark, the NiO‐modified surface showed minimal CO_2_ activation (such as weak CO* and other intermediates). Upon illumination, progressive enhancement of bridged Ni‐CO* and *COOH species were observed (solid‐arrow in Figure 4d), while the emergence of CO_2_^δ^^−^ species (1258 cm^−1^) indicated a light‐driven electron transfer pathways for CO_2_ reduction on the CeZrO_x_/NiO‐Ni catalyst (Figure 4c,d) [13, 30]. Relative to CeZrO_x_/Ni, illumination produced a reversal in *COOH/HCOO* intensities on the CeZrO_x_/NiO‐Ni, which can be ascribed to that CO_2_^δ^^−^ lowered the barrier to *COOH formation and accelerated the carboxylate route (Figure 4b‐d). Removing CO_2_ from the feed led to faster depletion of CO* and *COOH species than HCOO* on CeZrO_x_/NiO‐Ni (Figure S20), further supporting a carboxylate‐dominated pathway. The CO_2_^δ^^−^ band appeared only under illumination across two dark‐light cycles (Figure S21), confirming its light‐specific origin. Collectively, these observations indicate that NiO interfacial engineering enables a photochemical activation sequence (CO_2_^δ^^−^→*COOH→CO*→···→CH_x_*→CH_4_). Complementary CO_2_‐TPD showed preferential adsorption on weak‐to‐moderate basic sites for CeZrO_x_/NiO‐Ni, in contrast to strong‐site adsorption on NiO‐Ni/CeZrO_x_, suggesting more favorable CO_2_ activation (Figure S22).

Near‐ambient pressure X‐ray photoelectron spectroscopy (NAP‐XPS) was further used to probe the light‐response of CeZrO_x_/NiO‐Ni while avoiding vacuum‐induced reduction (Figure 4e; Figure S23). Under Ar, illumination produced a ∼0.2 eV shift of the Zr *3d* peak at ∼182.2 eV to lower binding energy, evidencing photo‐induced electron accumulation at the supported oxide surface. A similar light‑responsive shift was observed in the Zr *3d* peak under reactive atmosphere when the light was switched on and off, further confirming that irradiation drives interfacial electron transfer (Figure S24). Guided by these observations and the calculated band structure (Figure S8), the band alignment was proposed in Figure 4f. Under illumination, LSPR‐excited electrons in Ni are driven toward the oxide–metal interface. Given the lower work function of Ni relative to NiO, Schottky barrier can form at NiO‐Ni and hinder electron backflow [46, 47]. Contact between n‐type CeZrO_x_ and p‐type NiO creates a space‐charge region and internal field [48] that drives electrons from NiO to CeZrO_x_ and holes oppositely under illumination, matching the NAP‐XPS‐inferred carrier flow. Photocurrent measurements further support effective heterojunction operation, showing higher current densities and prolonged rise/decay transients upon light on and off, indicative of extended carrier lifetimes and improved interfacial transfer via the NiO layer (Figure S25). Compared with purely thermal operation at the same bed temperatures, photothermal illumination increased CO_2_ conversion from 8% to 25% at 160°C (0.59 W cm^−2^) and 49% to 83% at 200°C (0.71 W cm^−2^) (Figure 4g), demonstrating that overall activity arises from photothermal synergistic effect, facilitated by the NiO interface engineering that enhances photothermal conversion through intensified LSPR and tailored band structure.

## 3 Conclusions

In summary, the CeZrO_x_/NiO‐Ni inverse catalyst enabled effective photothermal CO_2_ methanation under mild irradiation via light‐induced self‐heating, achieving mol_CH4_ g_cat_^−1^ h^−1^ level of CH_4_ production rate in a flow reactor with CH_4_ selectivity exceeding 99%. The excellent performance of CeZrO_x_/NiO‐Ni was attributed to the enhanced LSPR effect and the formation of heterostructure, which promoted photothermal synergy by generating abundant hot electrons and activating photoelectron induced CO_2_ *^δ−^* →*COOH→CO* pathway under a low light intensity. This synergy enabled sustained CH_4_ production and high CO_2_ conversion, even under natural sunlight. Exceptional adaptability and operational durability were demonstrated through eight consecutive start‐stop cycles and full performance retention after three months of storage, attributable to the protective NiO layer. These findings indicate that the structurally robust and readily scalable Ni‐based inverse catalyst can operate effectively under intermittent renewable energy conditions, providing a catalyst‐level pathway for solar‐driven CO_2_ conversion when coupled with large‐scale solar collection and efficient H_2_ supply.

## 4 Experimental Section

Experimental details, including catalyst preparation, characterizations, and photothermal catalytic performance tests are provided in the Supporting Information.

## Funding

National Key R&D Program of China (No. 2024YFB4105300), Natural Science Foundation of China (No. 22502175, No. 22278367, No. 22272106), Fundamental Research Funds for the Provincial Universities of Zhejiang (No. RF‐C2025003, No. RF‐A2025005), Zhejiang Provincial Natural Science Foundation of China (No. LQ24B030016).

## Conflicts of Interest

The authors declare no conflict of interest.

## Supporting information




**Supporting File 1**: advs74196‐sup‐0001‐SuppMat.docx;

**Supporting File 2**: advs74196‐sup‐0002‐Data.zip.

## Data Availability

The data that support the findings of this study are available from the corresponding author upon reasonable request.
