# Supplementary material for: Efficient and Robust Heterostructure CeZrOx/NiO‐Ni Inverse Catalyst for Sustainable Photothermal CO2 Methanation
Source: Adv Sci (Weinh). 2026 Jan 30;13(20):e22942. doi: 10.1002/advs.202522942 (PMC13067768; doi:10.1002/advs.202522942)
Supplement: Supplementary file 1 — Supporting File 1: advs74196‐sup‐0001‐SuppMat.docx; [file ADVS-13-e22942-s002.docx]

Supporting Information

Efficient and Robust Heterostructure CeZrO_x_/NiO-Ni Inverse Catalyst for Sustainable Photothermal CO_2_ Methanation

Chuqiao Song^†12^, Zhaohua Wang^†34^, Zhouhong Ren^†9^, Cheng Yu^1^, Haibo Li^1^, Xin Tang^1^, Houhong Song^5^, Yao Xu^6^, Liangwei Liu^7^, Lili Han^7^, Liwei Chen^9^, Zhifu Qi^34^, Xi Liu*^89^, Siyu Yao^*5^, Xiao-nian Li^1^, Xiang Gao^*3^, Lili Lin^*12^

1 State Key Laboratory of Green Chemical Synthesis and Conversion, Zhejiang Key Laboratory of Surface and Interface Science and Engineering for Catalysts, College of Chemical Engineering, Zhejiang University of Technology, Hangzhou 310014, China

2 Zhejiang Carbon Neutral Innovation Institute & Zhejiang International Cooperation Base for Science and Technology on Carbon Emission Reduction and Monitoring, Zhejiang University of Technology, Hangzhou 310014, China

3 Science and Education Integration College of Energy and Carbon Neutralization, Zhejiang University of Technology, Hangzhou 310014, China

4 Zhejiang Baima Lake Laboratory Co., Ltd, Hangzhou 311121, China

5 State Key Laboratory of Chemical Engineering, College of Chemical and Biological Engineering, Zhejiang University, Hangzhou 310027, China

6 Beijing National Laboratory for Molecular Sciences, College of Chemistry and Molecular Engineering, Beijing 100871, China

7 State Key Laboratory of Structural Chemistry, Fujian Institute of Research on the Structure of Matter, Chinese Academy of Sciences, Fuzhou 350002, China

8 School of Chemistry and Chemical Engineering, Ningxia University, Yinchuan 750021, China

9 School of Chemistry and Chemical Engineering, In situ Center for Physical Sciences, Frontiers Science Center for Transformative Molecules, Shanghai Jiao Tong University, Shanghai 200240, China

† These authors contributed equally to this work.

* Email: liuxi@sjtu.edu.cn; xgaol@zju.edu.cn; yaosiyu@zju.edu.cn; linll@zjut.edu.cn.

**Methods**

**Chemicals and materials**

All reagents were commercially available and were used without any purification. Analytical grade chemicals including Ni(NO_3_)_2_·6H_2_O, Ce(NO_3_)_3_·6H_2_O, Zr(NO_3_)_4_·5H_2_O and other related nitrates were purchased from Macklin. H_2_C_2_O_4_·2H_2_O was purchased from Vetec.

**Catalyst preparation**

The CeZrO_x_/NiO-Ni catalysts were synthesized via a co-precipitation method (CP), using oxalic acid as the precipitating agent, followed by calcination, reduction and passivation. The mole percent of CeZrO_x_ was 0, 5, 13, 30, 50, 87, 100%, including pure NiO (0 mol% CeZrO_x_), pure CeZrO_x_ (100 mol% CeZrO_x_), inverse catalyst (5, 13, 30 mol% CeZrO_x_), and conventional supported catalyst (50, 87 mol% CeZrO_x_). In general, the CeZrO_x_ loading of the most frequently mentioned CeZrO_x_ /(NiO-)Ni inverse catalyst in the manuscript is 13 mol%, and the Ni loading of the (NiO-)Ni/ CeZrO_x_ conventional catalyst is 13 mol%. Taking the synthesis procedure of 13 mol% CeZrO_x_/NiO-Ni catalyst as an example: 10 mmol of Ni (NO_3_)_2_·6H_2_O, 0.75 mmol Zr(NO_3_)_4_·5H_2_O and 0.75 mmol Ce(NO_3_)_3_·6H_2_O precursors were dissolved in 50 mL ethanol. The mixed solution of precursors was then added into 0.5 M oxalic acid in ethanol solution under vigorous stirring. After 30 min reaction, the resultant solid was separated by centrifugation, followed by washing with ethanol and drying overnight. The obtained solid was calcined in the furnace at 400 °C for 2h, and the obtained solid powder was marked as CeZrO_x_/NiO. The CeZrO_x_/NiO catalyst was reduced in a stream of 20% H_2_/N_2_ at 450 °C for 3 h under atmospheric pressure, and the obtained solid powder was marked as CeZrO_x_/Ni. Then, the reduced catalyst was passivated in a stream of 0.5% O_2_/N_2_ at 25 °C for 8 h under atmospheric pressure, obtaining the final CeZrO_x_/NiO-Ni catalysts.

The loadings of Ce, Zr and Ni were determined by ICP-OES.

**Photothermal methanation of CO_2_**

The photothermal CO_2_ hydrogenation reaction was carried out in a 50 mL flow reactor (Quzhou Vodo Instrument Co., LTD) equipped with a heating chamber and quartz window. In a typical test, 40-80 mg catalyst was laid flat on the cellular reactor lining, where the reaction gas (CO_2_:H_2_:N_2_ =18:72:10) can flow through. The total gas flow rate was controlled at 12-200 mL/min for different WHSV. A 300 W Xe lamp (320-1000 nm, PLS-SXE300, Beijing Perfect light) was used as the illumination source, and the light intensity was detected by an optical power meter (PL-MW2000, Beijing Perfect light). The temperature of catalyst under light irradiation were tested by an IR camera and a K-type thermocouple inserted into the bad of catalysts. The outlet gas of the reactor was analyzed by an online gas chromatographer (GC-8860, Agilent), which was equipped a thermal conductivity detector (TCD) and a flame ionization detector (FID). The CO_2_ conversion and CH_4_ selectivity were calculated based on the GC data. The CO_2_ conversion, CH_4_ selectivity and space time yield (STY) of CH_4_ were defined in the following equation:

$$\text{Conv.}\left( \text{CO}_{\text{2}} \right)\text{\%=}\frac{\text{F(}\text{CO}_{\text{2}}\text{)*}\text{C}_{\text{in}}\left( \text{CO}_{\text{2}} \right)\text{-F}\text{CO}_{\text{2}}\text{*}\frac{\text{A}_{\text{N}_{\text{2}}\text{-in}}}{\text{A}_{\text{N}_{\text{2}}\text{-out}}}\text{*C}_{\text{out}}\left( \text{CO}_{\text{2}} \right)}{\text{F}\text{(CO}_{\text{2}}\text{)*}\text{C}_{\text{in}}\left( \text{CO}_{\text{2}} \right)}$$

$$\text{Sel.(}\text{CH}_{\text{4}}\text{)=}\frac{\text{n}\text{CH}_{\text{4}}}{\text{nCO+n}\text{CH}_{\text{4}}}$$

$$\text{STY(}\text{CH}_{\text{4}}\text{)(}\text{mmol}_{\text{CH}_{\text{4}}\text{∙}}\text{g}_{\text{cat∙}}^{\text{-1}}\text{h}^{\text{-1}}\text{)=}\frac{\text{F}\text{CO}_{\text{2}}\text{*Conv.(}\text{CO}_{\text{2}}\text{)*Sel.(}\text{CH}_{\text{4}}\text{)*60}}{\text{22.4*}\text{m}_{\text{cat}}}$$

$$\text{Carbon balance \%=}\frac{\text{∑n(products)}}{\text{n}_{\text{in}}\left( \text{CO}_{\text{2}} \right)\text{-}\text{n}_{\text{out}}\left( \text{CO}_{\text{2}} \right)}$$

F: the gas flow into the reactor; C: the concentration; A: the gas chromatographic peak area; m: the weight of catalyst; n: the amount of substance.

The outdoor performance test was carried out in a home-made flow reactor in Deqing County, Huzhou City, Zhejiang Province, from 10:00 to 15:00 on January 7, 2025. The weather was sunny with temperatures ranging from 0 to 9 °C and a northerly wind of force 2 (https://mirror-earth.com).The sunlight was concentrated to parallel light through a Fresnel lens and plan-convex lens. The reacted gas was collected in gas collecting bags and then detected by gas chromatography. The results shown in Figure 3g correspond to product analysis from gas-bag sampling performed between 12:00-13:00. Light intensity was monitored using a optical power meter, and between 12:00-13:00 it varied from 0.80 to 1.10 W cm^-2^.

All the photothermal catalytic performance tests were conducted under ambient pressure and without external heating. The control experiments of thermocatalytic performance tests were carried out in the same reactor, using the built-in heating module. The setting temperature of thermocatalytic performance test was same with the temperature measured under illumination, and other test conditions were consistent with photothermal catalytic test.

**Characterization**

***Finite difference time domain (FDTD) method.*** The electric field distribution of the nanostructures was computed using the finite difference time domain (FDTD) method. The dielectric functions of Ni and NiO were taken from the literature.^[1,2]^ To mitigate unphysical scattering, perfectly matched layer (PML) boundary conditions were applied in all x, y, and z directions. For enhanced accuracy, the simulation domain was discretized into a mesh with a resolution of 0.5 nm × 0.5 nm × 0.5 nm. The side length of the simulation domain was set to 5–10 times the excitation wavelength to avoid boundary effects. A total-field/scattering-field plane wave was employed as the excitation source, incident perpendicularly onto the nanostructure surface, with continuous illumination rather than a pulsed source. An electric field monitor was then used to capture the resulting field distribution within the nanostructures. The convergence criterion was set to 1 × 10^-5^ fs, ensuring numerical stability and accuracy of the results.

***Inductively Coupled Plasma-Optical Emission Spectrometer.*** ICP-OES measurements were carried out on a Prodigy 7 Inductively Coupled Plasma-Atomic Emission Spectrometer (Leeman Ltd.). The samples (~1 mg) were digested with 0.75 mL hydrochloric acid, 0.25 mL nitric acid and 0.1 mL hydrofluoric acid at 260℃ in an UltraWAVE ECR Microwave Digestion System (Milestone Ltd.).

***BET analysis.*** N_2_ physisorption was performed on a Micromeritics ASAP-2020 instrument. The samples were first degassed in vacuum at 200 °C for 1 h before the measurement. The specific surface area and pore size distribution were calculated by using the Brunauer-Emmett-Teller (BET) method and Barrett-Joyner-Halenda (BJH) desorption branch, respectively.

***X-ray diffraction.*** X-ray diffraction (XRD) patterns were performed on PANalytical X’Pert PRO powder diffractometer with Cu Kα radiation (λ= 0.1541 nm), 40 kV working voltage and 40 mA working current. The 2θ range for pattern collection were collected from 10° to 80° with a step of 0.0167°. According to the strongest h k l (111) diffraction peak of Ni, the average crystallite sizes of the samples were calculated with the Scherrer equation.^[3]^

***X-ray photoelectron spectroscopy.*** The quasi *in-situ* X-ray photoelectron spectroscopy (XPS) characterizations were performed on a ThermoFischer ESCALAB 250Xi equipped with an *in-situ* reactor, using Al Kalpha radiation as excitation source (hv = 1486.6 eV), 8x10^-10^ mbar analysis chamber vacuum level, 12.5 kV working voltage, and 16 mA filament current. The passing energy was 30 eV with a step of 0.1 eV, and the signal accumulation was approximately 10 cycles. Before quasi *in-situ* XPS testing, the catalyst sample was placed inside the reactor chamber and pre-treated by 20% H_2_ at 450 °C for 3 h. The sample was transferred to the measurement chamber without ingress of air after cooling to room temperature. Then, the sample was transferred back to the reactor chamber, treated by 0.5% O_2_ at 25 °C for 8 h, transferred to the measurement chamber without ingress of air after cooling to room temperature again. The measurement chamber was evacuated to a vacuum level below 8x10^-10^ mbar before conducting the analysis. The XPS spectra were processed using CasaXPS software and 284.8 eV was used as the energy calibration reference.

***Near ambient pressure XPS****.* The NAP-XPS analysis was conducted using a laboratory backfilling NAP-XPS system provided by SPECS GmbH, equipped with a PHOIBOS 150NAP, 1D-DLD analyzer and an Al K_α_ (1486.7 eV) quartz monochromator source. The pre-catalyst was pressed into a small pellet with a diameter of 6 mm and placed on a sample holder made of tantalum. The pretreated sample was first transferred to the analysis chamber and equilibrated under 1 mbar of Ar at room temperature. It was then subjected to xenon lamp irradiation, with repeated light-on/light-off tests. Subsequently, the atmosphere was switched to a CO_2_/H_2_ mixture (1:4 ratio), and the light-cycling tests were repeated under identical irradiation conditions. Corresponding spectra were collected at each stage.

***In-situ Diffuse reflectance infrared flourier transform spectroscopy.*** *In-situ* Diffuse reflectance infrared flourier transform spectroscopy (DRIFTS) were performed on Bruker Vertex 70V, which was equipped a Harrick cell and a liquid-nitrogen-cooled MCT detector. Before testing, the catalysts were pre-treated by 20% H_2_ (10 mL min^-1^, H_2_/Ar = 2:8, 0.1 MPa) at 450 °C for 3 h, then cooled down to 150 °C and purged with pure Ar for 30 min. The spectra were acquired with a resolution of 4 cm^-1^, and the acquisition time of each spectrum was about 1min. Background was collected first at pure Ar and test temperature prior to each experiment. The test temperature was chosen to 150 ℃, in order to better observe the intermediate species at a lower activity. The transmittance was obtained by dividing the collected sample reflectance spectrum by the background spectrum, then spectrum was converted to Kubelka-Monk (KM). Then the gas flow was changed to H_2_/CO_2_ (10 mL min^-1^, H_2_/CO_2_ = 8:2, 0.1 MPa) at the same temperature and the spectra were collected simultaneously. After 60 min reaction in H_2_/CO_2_ atmosphere, the gas flow was changed to H_2_/Ar (10 mL min^-1^ H_2_/Ar = 8:2, 0.1 MPa), keeping the temperature constant for another 60 min. During the entire process, DRIFTS spectra were recorded to monitor the change of intensity of different surface species on catalysts. Additional light was introduced by an optical fiber for the spectrum with illumination, and other test conditions were consistent with under heating conditions.

***Raman analysis*.** Both *ex-situ* and *in-situ* Raman spectroscopy were conducted. For *ex-situ* Raman measurements, spectra were recorded at room temperature using a Horiba LabRam HR Evolution spectrometer, which was equipped with a He-Ne laser (λ = 532 nm) operating at 10 mW, a CCD detector, and an Olympus microscope fitted with a 50× objective lens. *In-situ* Raman measurements were performed using a custom-built optical Raman cell. The samples were first reduced in a 25 mL/min 20% H_2_/Ar mixture at 723 K for 3 hours. After passivation, the gas flow was switched to 20 mL/min 0.5% O_2_/Ar. *In-situ* Raman spectra were collected with two accumulations of 30 s each under varying atmospheric conditions.

***STEM and EDS characterization.*** STEM and EDS mapping measurements were conducted using an aberration-corrected scanning transmission electron microscope (Hitachi HF5000). Additionally, a probe-corrected ESTEM system, equipped with dual Oxford Instruments XEDS detectors (2 × 100 mm^2^), was employed and operated at an accelerating voltage of 200 kV. This instrument facilitated simultaneous STEM imaging using a bright field (BF) detector, an annular dark field (ADF) detector, and a secondary electron (SE) detector. With the aid of ADF and SE imaging, both bulk and surface microstructures were resolved at atomic resolution (>0.7 Å) in both STEM-ADF and STEM-SE images.

***CO_2_-temperature-programmed desorption*.** CO_2_-TPD tests were performed on a Bel mass spectrometer. Before testing, the samples were pre-treated by 20% H_2_ (10 mL min^-1^, H_2_/Ar = 2:8, 0.1 MPa) at 450 °C for 3 h, then purged with Ar for 30 min, and cooled down to room temperature. The passivated sample was subsequently treated with 0.5%O_2_/N_2_ for another 8 h at room temperature. CO_2_ adsorption was performed in 10% CO_2_ (10 mL min^-1^, CO_2_/Ar = 1:9, 0.1 MPa) for 60 min, then the samples were purged with Ar for 40 min to remove the un-adsorbed CO_2_. Then, the CO_2_ desorption was performed with heating rate of 10 °C/min, detected by a mass spectrometer.^[3]^

***Optical properties analysis.*** Ultraviolet visible (UV-vis) diffuse reflection spectra and photoluminescence (PL) spectra were analyzed on a spectrophotometer (SHIMADZU UV-3600, Japan). The Mott-Schottky measurement was performed at frequency of 2000 Hz. Photocurrent tests were carried out using a 300 W Xe lamp.

**Statistical analysis**

The error bars represent the deviation of three independent replicate experiments. Data analysis and graphical visualization for the performance tests were conducted using OriginPro 2020b (OriginLab). The analytical methods employed for spectroscopic characterization are described in detail in the corresponding characterization section.


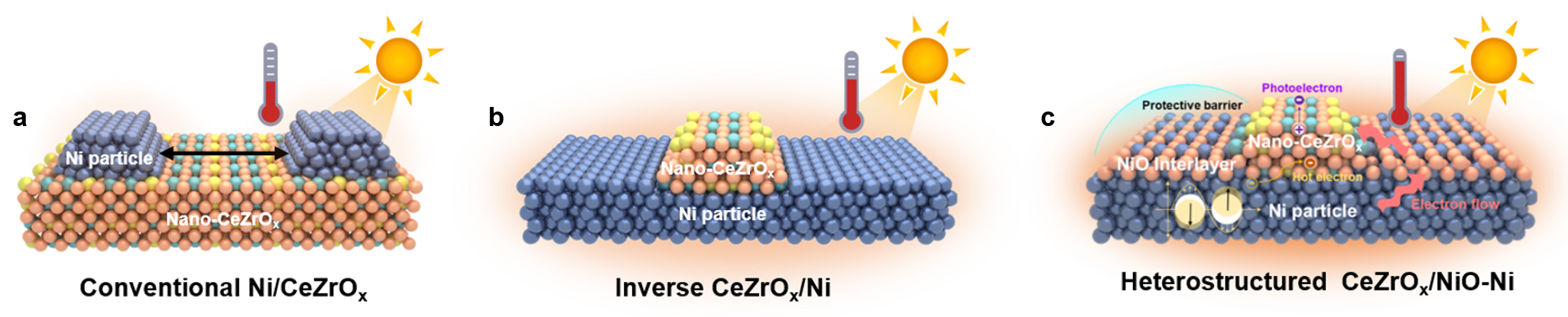


**Figure S1.** Schematic diagram of **a.** Ni/CeZrO_x_ conventional catalyst, **b.** CeZrO_x_/Ni inverse catalyst, and **c.** heterostructured CeZrO_x_/NiO-Ni inverse catalyst.


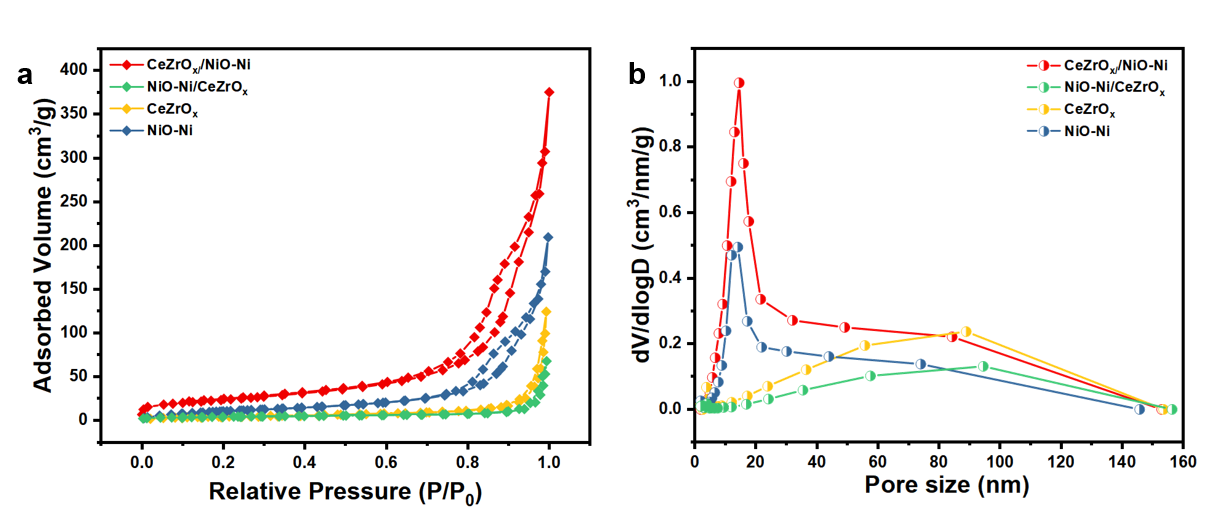


**Figure S2.** BET results of different Ni-based and CeZrO_x_ catalysts. **a.** N_2_ adsorption/desorption isotherm. **b.** Pore size distribution. The specific data were presented in **Table S1**.


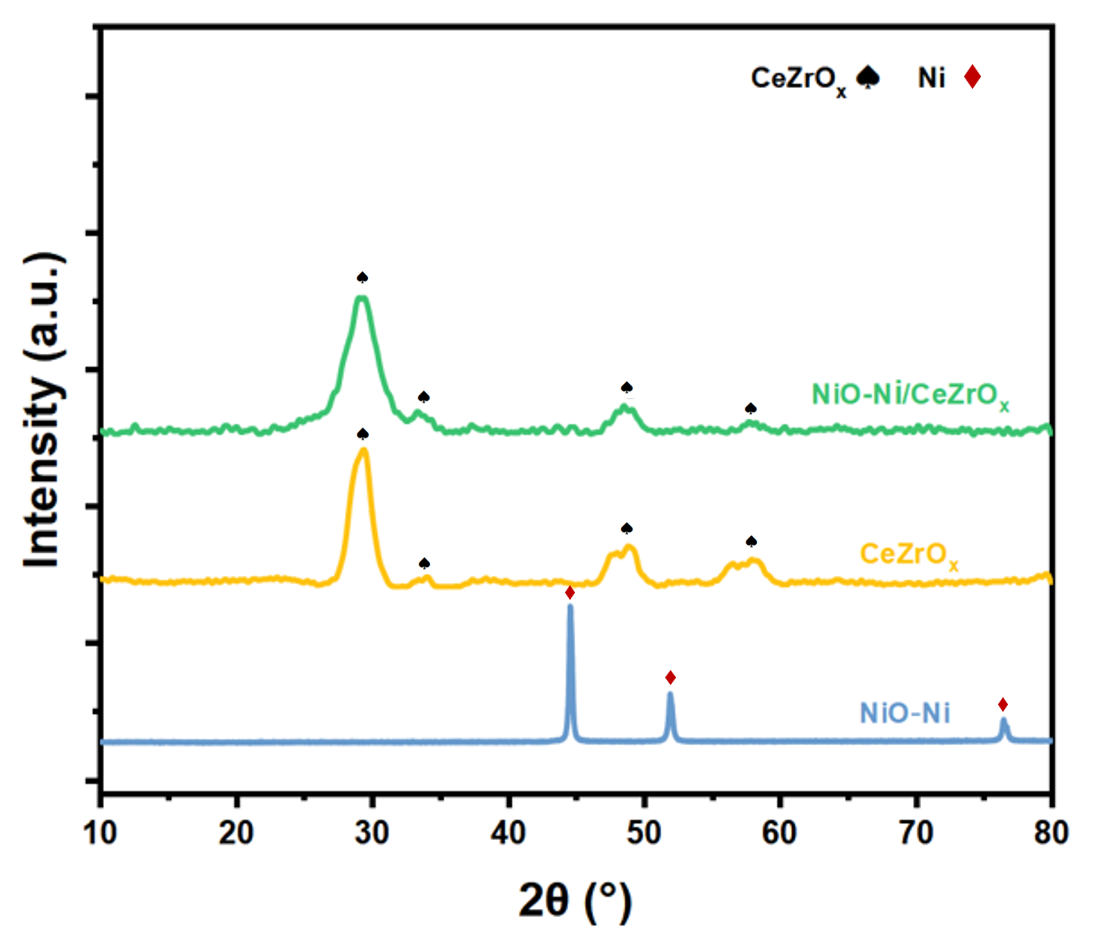


**Figure S3.** XRD patterns of NiO-Ni (metallic Ni after passivation), CeZrO_x_ and NiO-Ni/CeZrO_x_ catalysts.


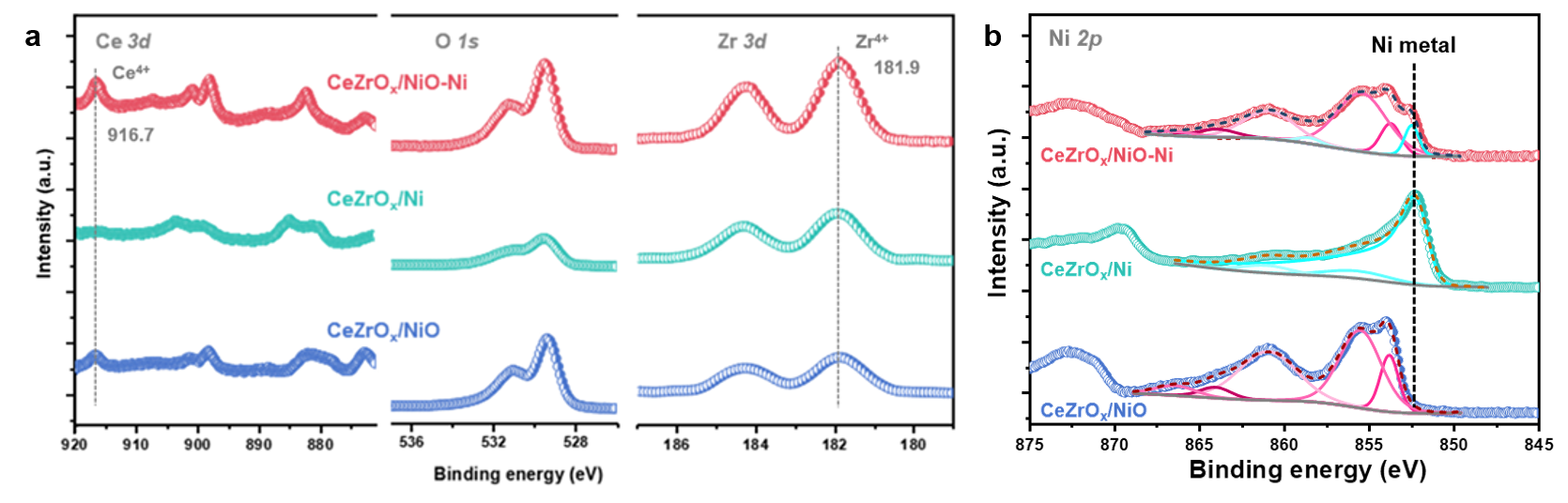


**Figure S4.** Quasi *in-situ* XPS of **a.** Ce *3d*, O*1s*, Zr *3d* and **b.** fitted Ni *2p* XPS spectra of the CeZrO_x_/NiO-Ni catalyst in the synthesis process.


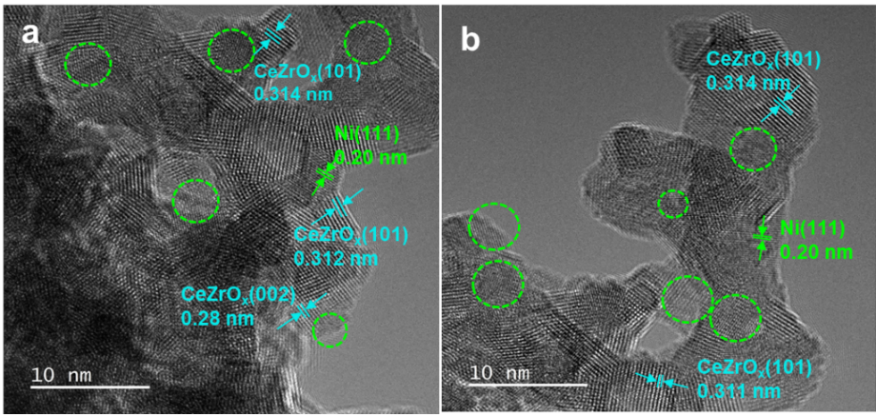


**Figure S5.** HAADF-STEM images of 13 mol% NiO-Ni/CeZrO_x_ (87 mol% CeZrO_x_/NiO-Ni) conventional catalyst.


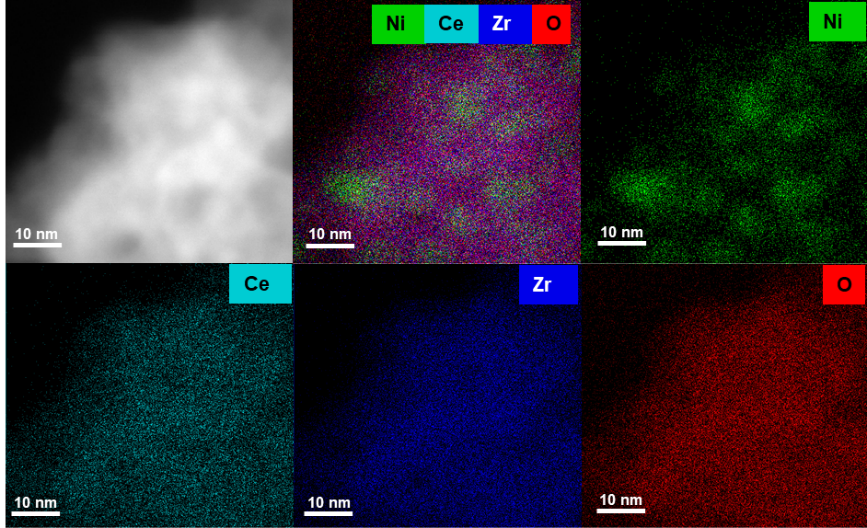


**Figure S6.** EDS elemental mappings of NiO-Ni/CeZrO_x_ conventional catalyst (green: Ni, dark blue: Zr, red: O, light blue: Ce; and superposition of colors of O, Zr and Ce).


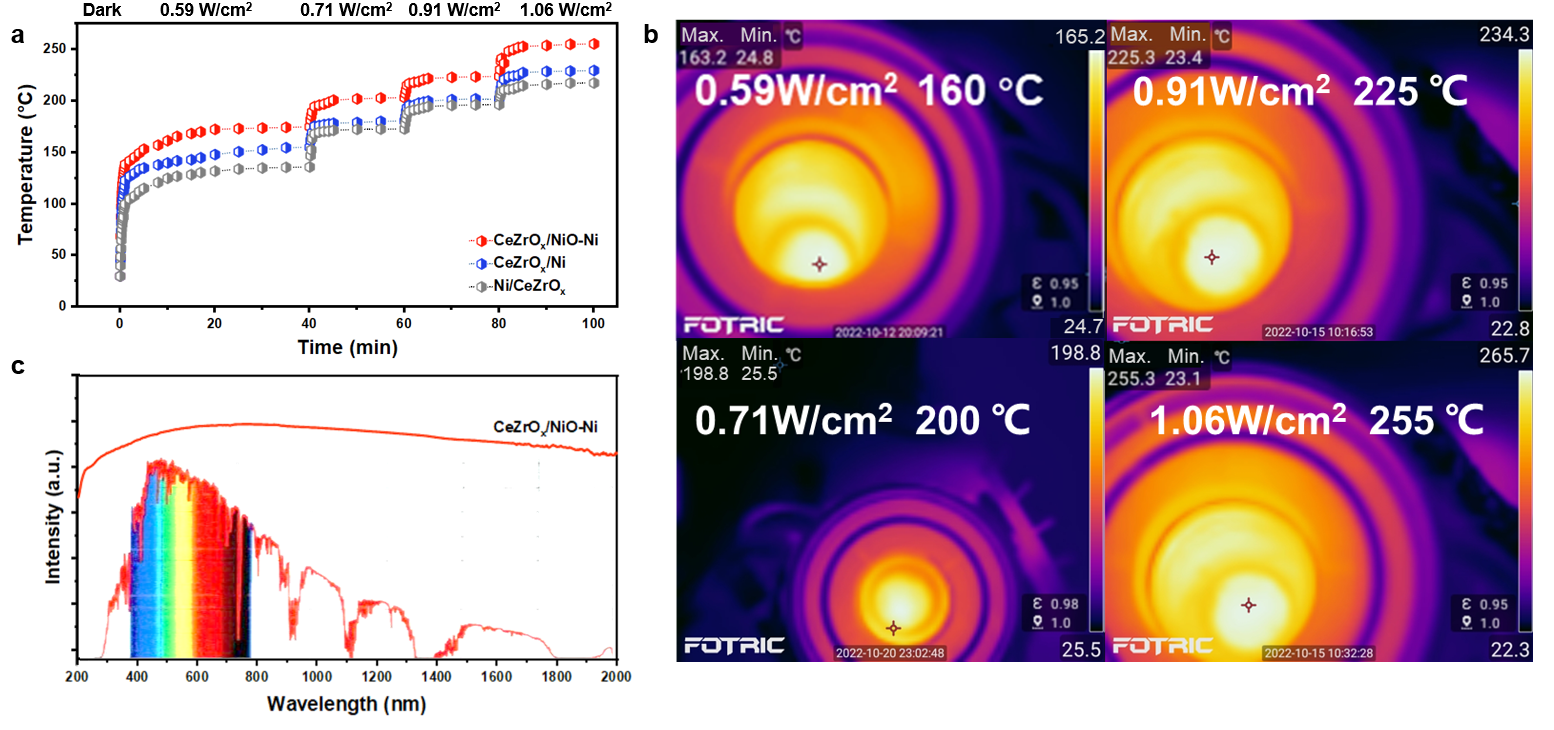


**Figure S7.** Photothermal properties of the CeZrO_x_/NiO-Ni and CeZrO_x_/Ni catalysts. **a.** Temperature of the catalysts bed under irradiation with different light intensities. **b.** Thermal imaging under corresponding light intensities on the CeZrO_x_/NiO-Ni. **c.** Light absorption across the full spectrum on the CeZrO_x_/NiO-Ni, with the solar spectrum presented as well.

The catalyst bed temperature was measured in an N_2_ atmosphere. Under actual reaction conditions, however, no significant temperature rise was observed, since the flat catalyst bed effectively suppresses the formation of reaction hot spots.


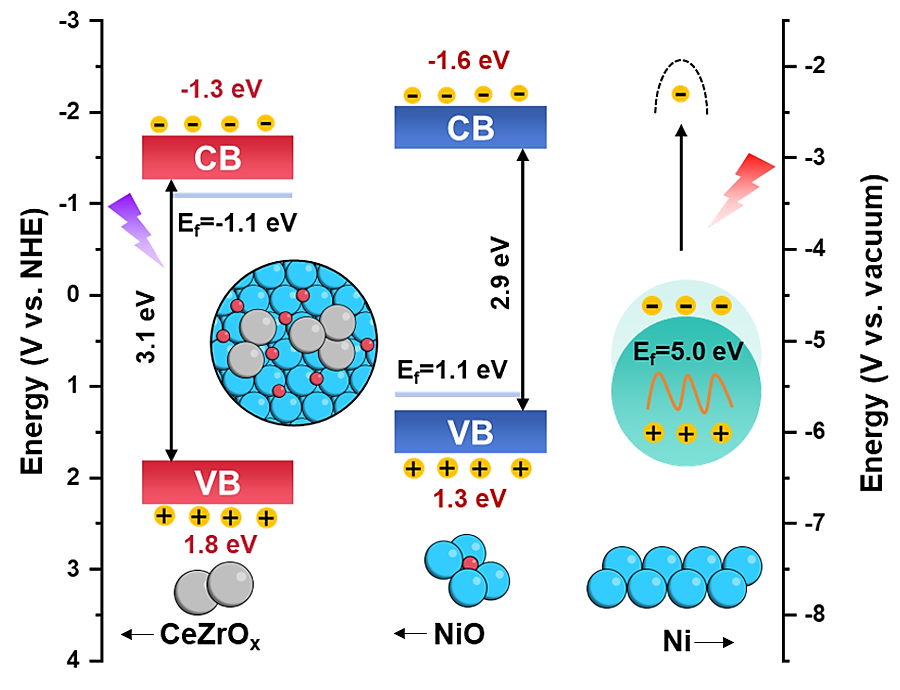


**Figure S8.** Energy band diagram of CeZrO_x_, NiO and metallic Ni^[4]^.

The flat potentials of CeZrO_x_ and NiO were -1.1V and 1.1V versus normal hydrogen electrode (NHE) according to **Figure 2g**, and it is generally accepted that the value of the flat potentials is approximately equal to that of the Fermi level. Consequently, according to the empirical value (~0.2 eV), the valence band (CB) of CeZrO_x_ can be calculated as -1.3 V, and the conduction band (VB) of NiO was 1.3 eV. The energy levels of CeZrO_x_ and NiO were plotted versus NHE, and the work function of metallic Ni was shown relative to vacuum. The relative positions of the three samples in the diagram are calibrated.


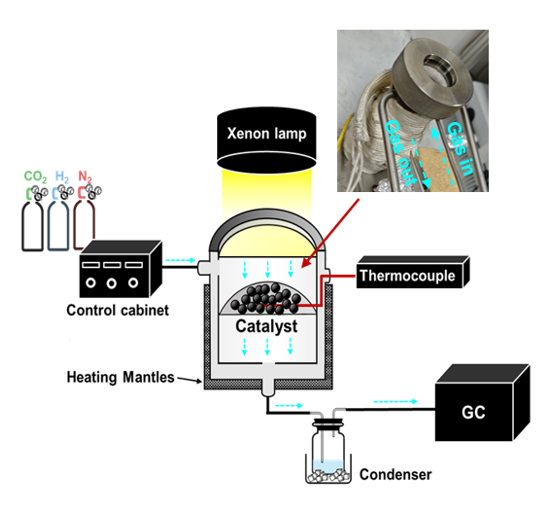


**Figure S9.** Schematic images of the photothermal reactor with corresponding practical photos.


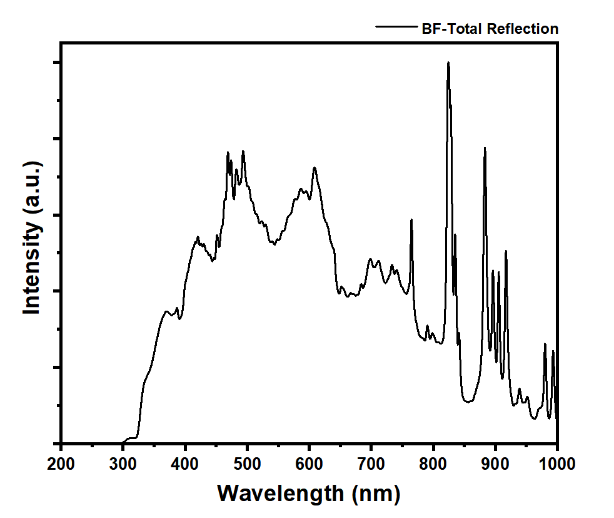


**Figure S10.** Spectrum of the 300 W Xe lamp light.


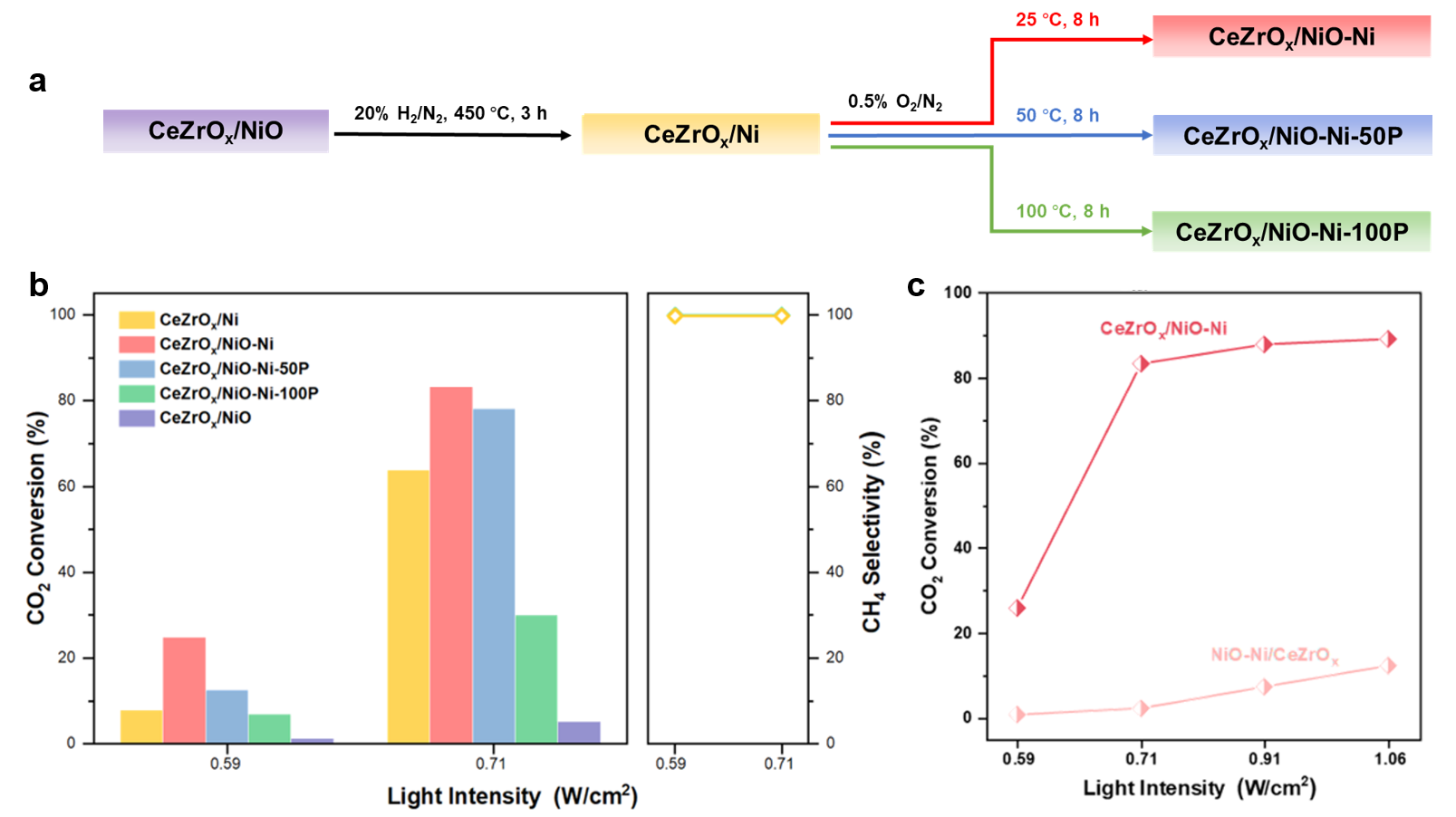


**Figure S11. a.** Schematic of catalysts preparation. **b.** CO_2_ conversion under different light intensities of CeZrO_x_/NiO-Ni catalysts with different oxidation degrees. **c.** Comparison of the CO_2_ conversion between inverse catalyst of CeZrO_x_/NiO-Ni and NiO-Ni/CeZrO_x_ with different light intensities. Reaction conditions: WHSV=9000 mL g_cat_^-1^ h^-1^, 300 W Xenon lamp, no external heating, CO_2_:H_2_:N_2_= 18:72:10, P = 0.1 MPa.

CeZrO_x_/NiO-Ni catalysts with controlled oxidation degrees were prepared at passivation temperatures of 25, 50, and 100 °C, labeled in **Figure S11a** as CeZrO_x_/NiO-Ni, CeZrO_x_/NiO-Ni-50P, and CeZrO_x_/NiO-Ni-100P, respectively. The CeZrO_x_/NiO-Ni catalyst passivated at 25 °C exhibited optimized catalytic activity.


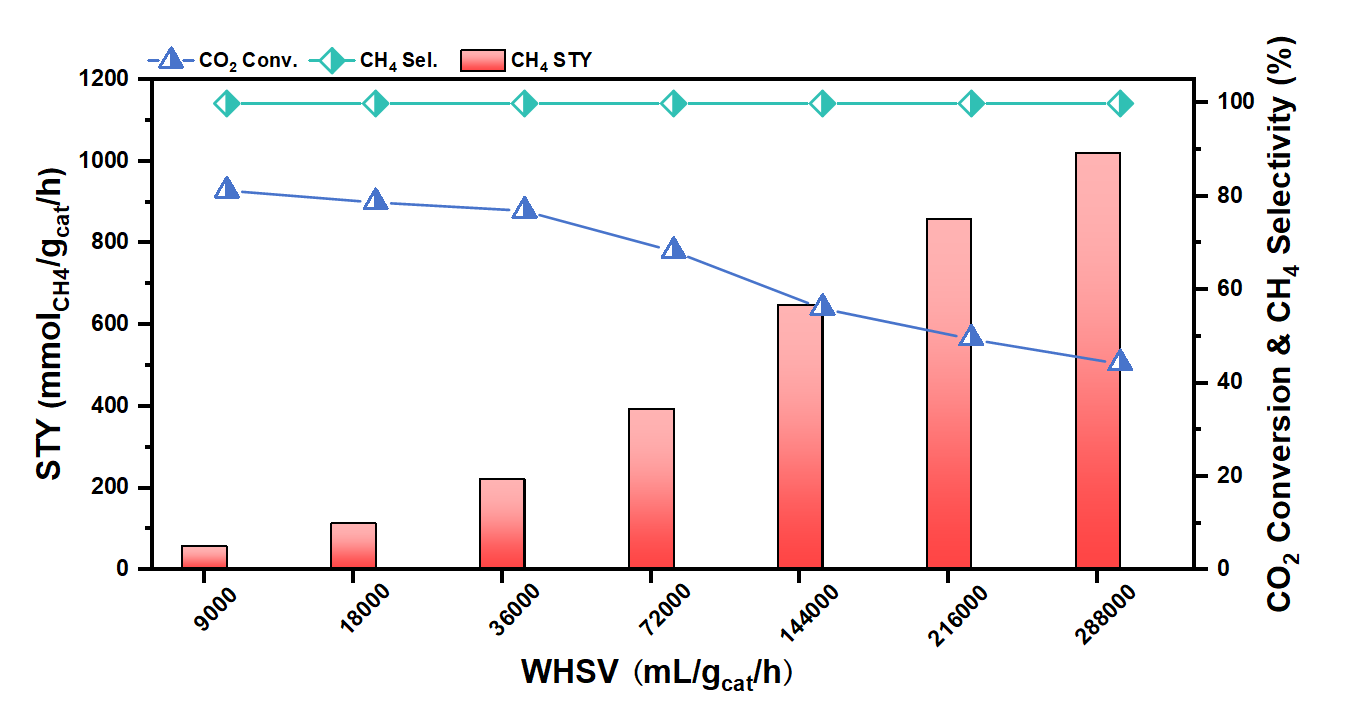


**Figure S12.** Photothermal catalytic activity of 87mol_Ni_% CeZrO_x_/NiO-Ni as a function of the WHSV (from 9000 to 288,000 mL g_cat_^-1^ h^-1^) under light intensities of 0.71 W/cm^2^.


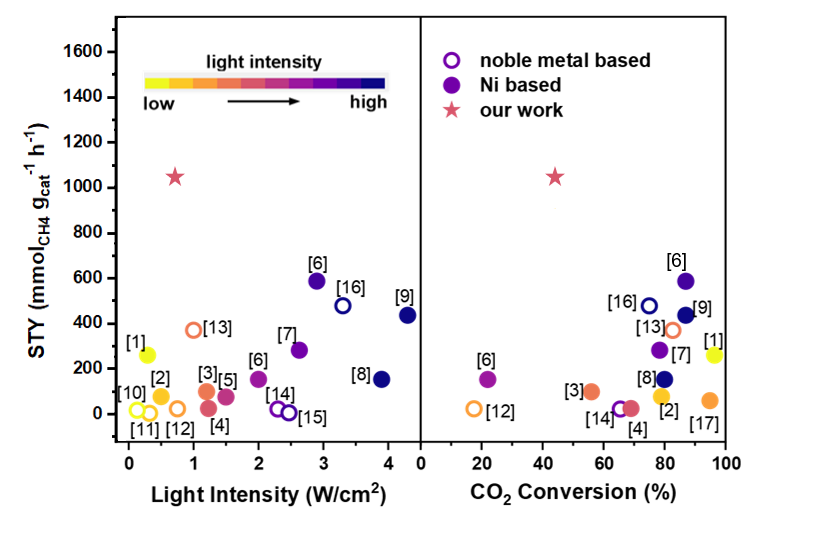


**Figure S13.** Performance (CH_4_ STY) comparison on light intensity and CO_2_ conversion between the current catalyst and previously reported photothermal catalysts. [1]^[5]^[2]^[6]^[3]^[7]^[4]^[8]^[5]^[9]^[6]^[10]^[7]^[11]^[8]^[12]^[9]^[13]^[10]^[14]^[11]^[15]^[12]^[16]^[13]^[17]^[14]^[18]^[15]^[19]^[16]^[20]^[17]^[21]^.


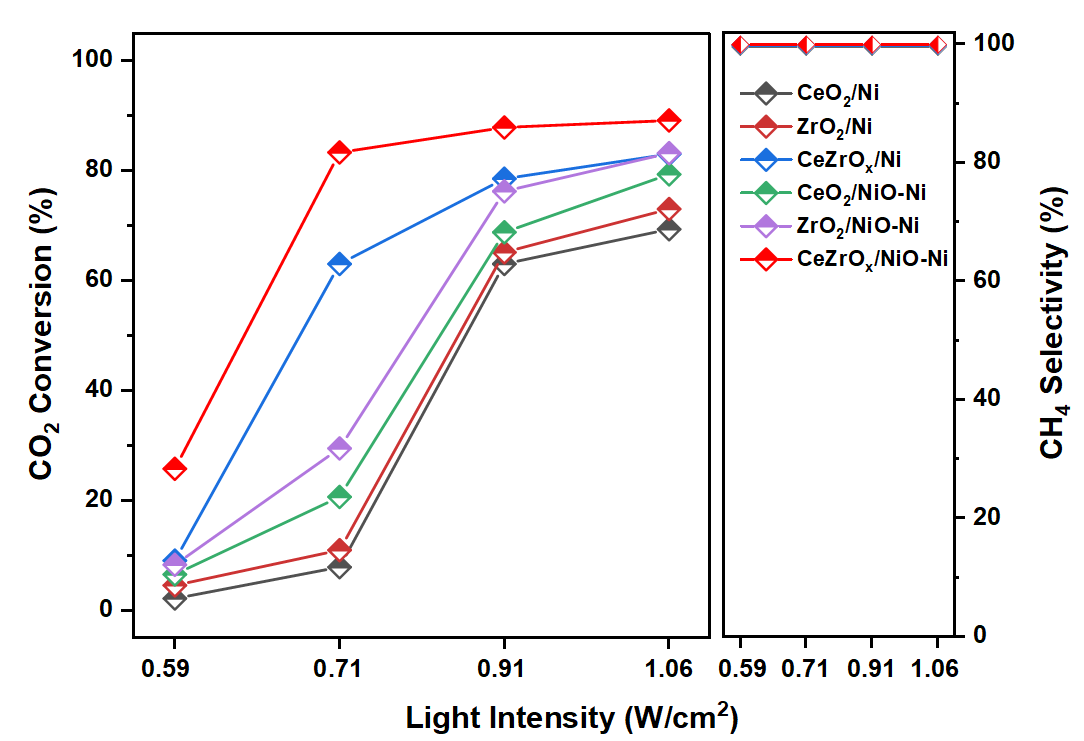


**Figure S14.** Performance of photothermal CO_2_ hydrogenation on Ni-based inverse catalysts using various oxides. Reaction conditions: WHSV=9000 mL g_cat_^-1^ h^-1^, 300 W Xenon lamp, no external heating, CO_2_:H_2_:N_2_= 18:72:10, P = 0.1 MPa.


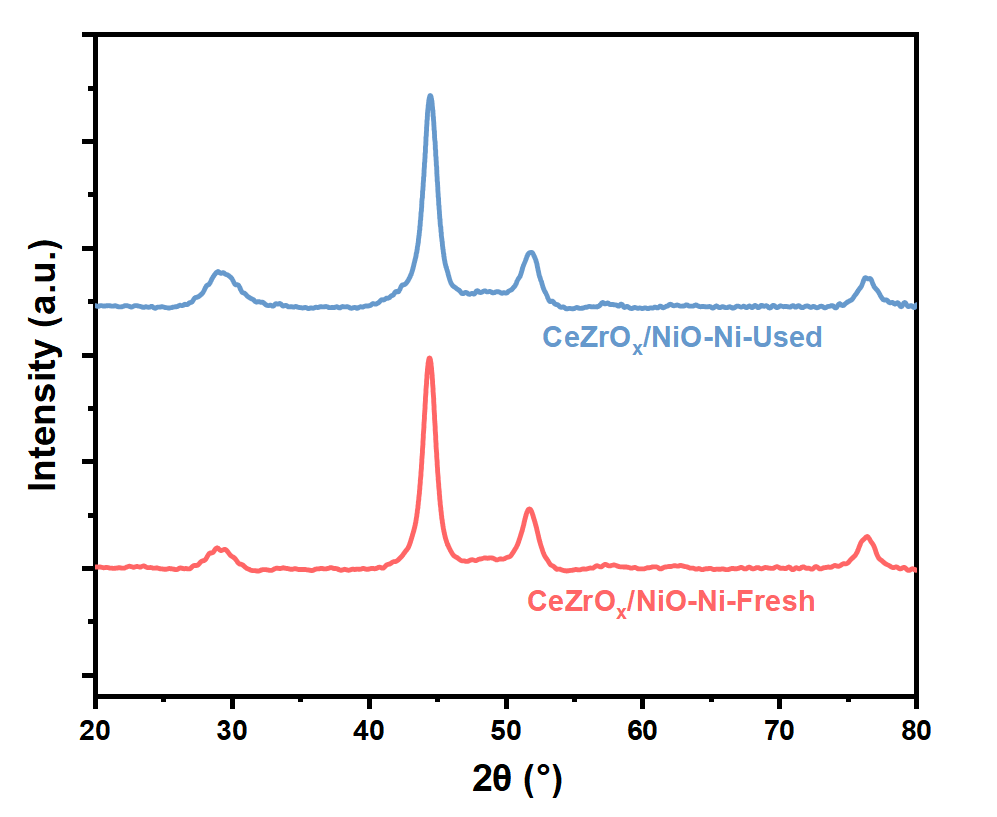


**Figure S15.** XRD patterns of CeZrO_x_/NiO-Ni before and after 50 h and 8 times of light on-off cycles test.


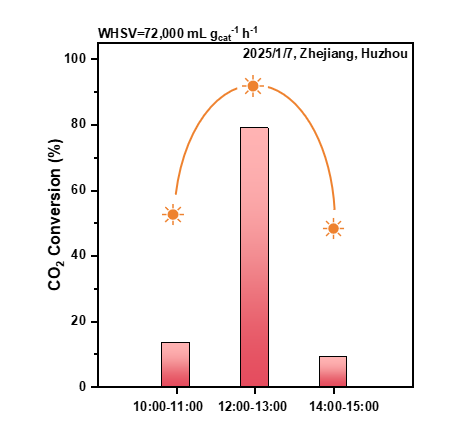


**Figure S16.** CO_2_ conversion for the photothermal process under outdoor sunlight irradiation.

The outdoor experiment was conducted in Deqing County, Huzhou City, Zhejiang Province, from 10:00 to 15:00 on 2025/01/07. The weather was sunny with temperatures ranging from 0 to 9 °C and a northerly wind of force 2 (https://mirror-earth.com).


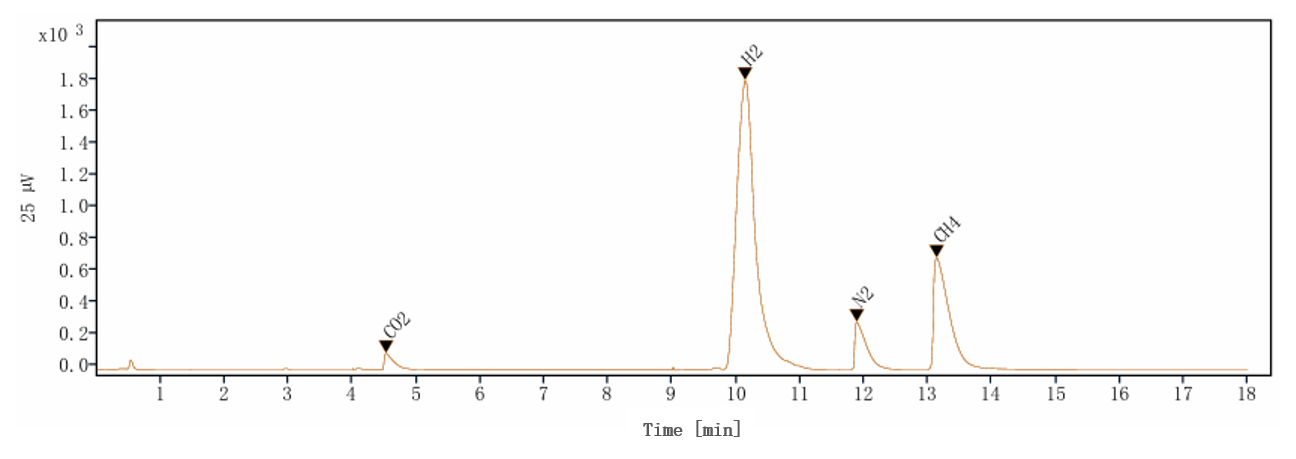


**Figure S17.** TCD signal on gas chromatography of the performance test under sunlight in Zhejiang, Huzhou, 2025/01/07, 12:00-13:00.


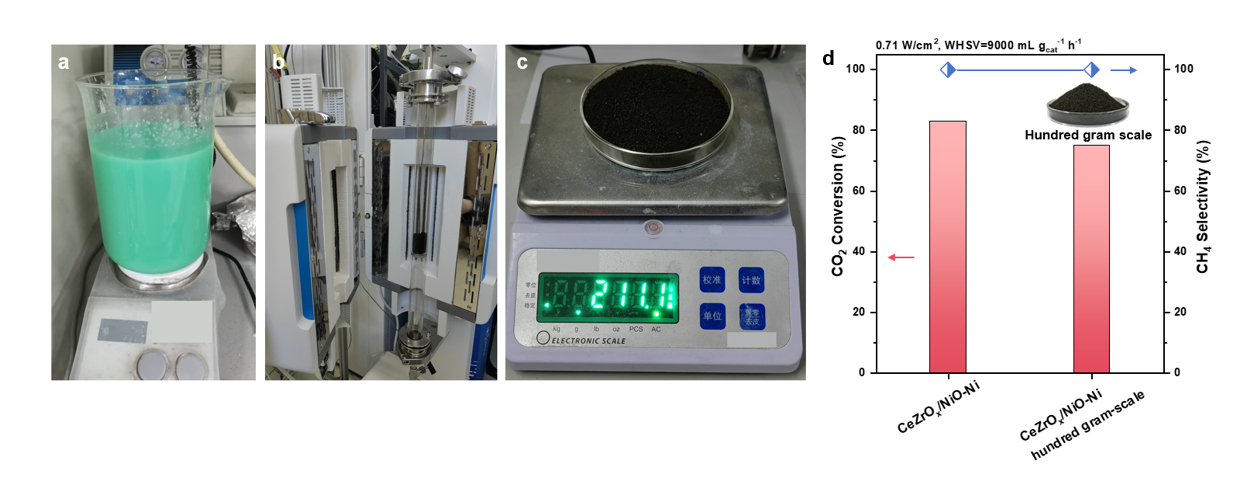


**Figure S18.** The device of inverse catalyst **a.** amplification synthesis, **b.** atmosphere treatment and **c.** real picture, **d.** CO_2_ conversion and selectivity on the CeZrO_x_/NiO-Ni in laboratory scale and 100-gram scale. The inset image was the picture of the catalysts in 100-gram scale.


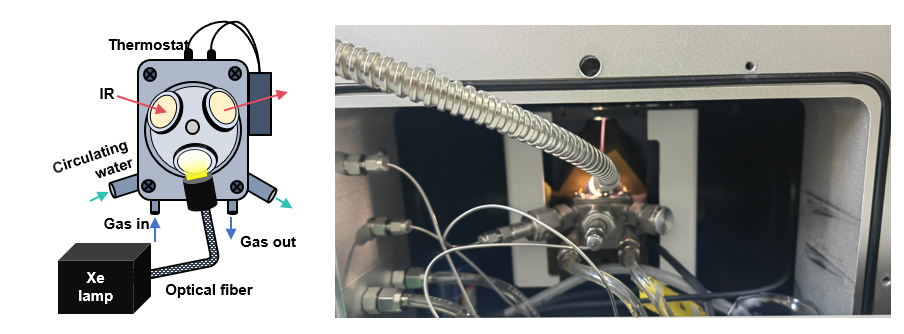


**Figure S19.** The *in-situ* cell and optical fiber of DRIFTS.


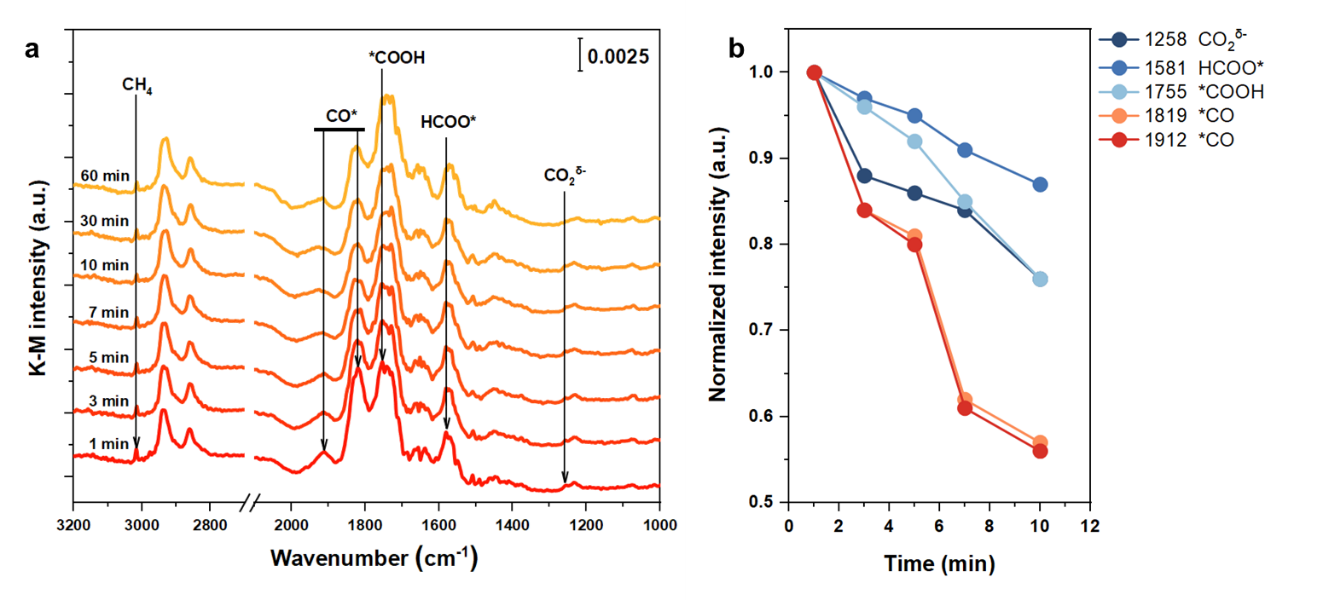


**Figure S20. a.** *In-situ* DRIFTS spectra of the H_2_ atmosphere on CeZrO_x_/NiO-Ni catalyst (pretreat 60 mins in 80% H_2_/20% CO_2_ atmosphere at 150 ℃ with illumination and the inlet was switched to 80% H_2_/20% Ar and maintained at the same condition for 60 min); **b.** Normalized intensities of the typical surface species in Figure S18a versus reaction time, the intensities of the species were normalized to each corresponding IR band after 1min reaction in 80% H_2_/20% Ar atmosphere.


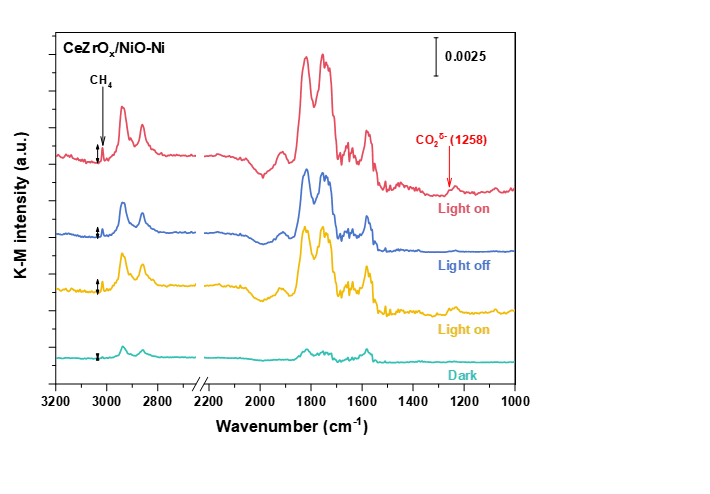


**Figure S21.** *In-situ* DRIFTS results on the CeZrO_x_/NiO-Ni under CO_2_/H_2_/N_2_= 18:72:10 at 150 ℃ with the switches from light-dark.

The spectrum diagram was collected after being maintained in the reaction gas for 60 minutes to ensure the stability of the intermediate species.


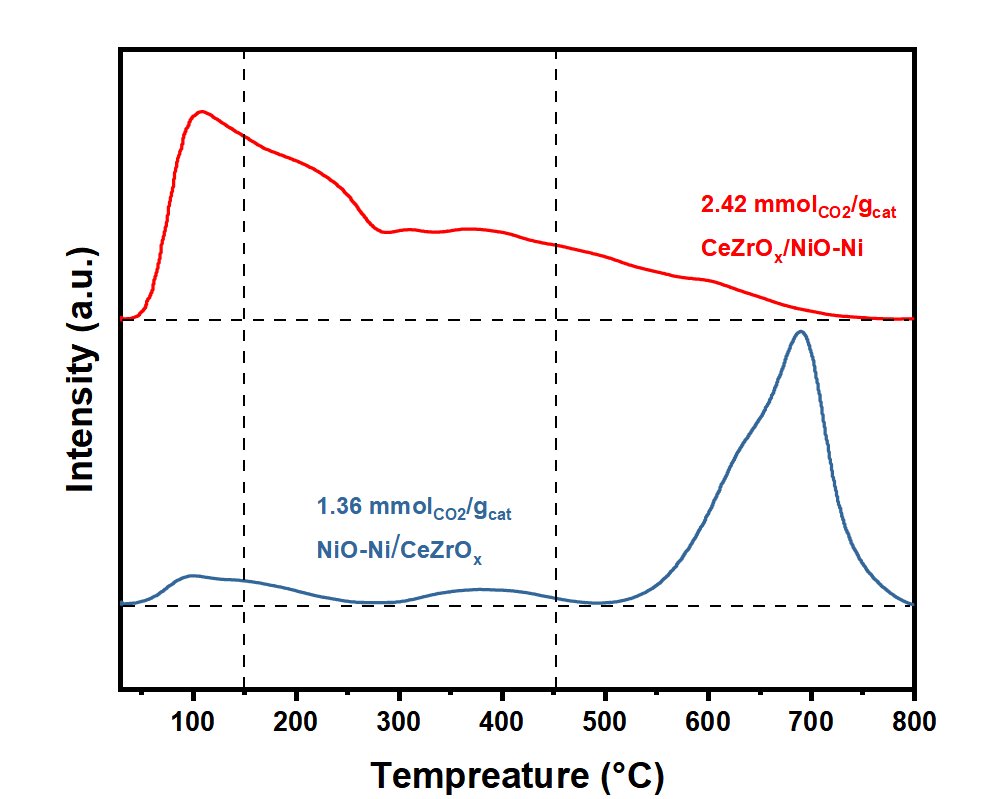


**Figure S22.** CO_2_-TPD results for CeZrO_x_/NiO-Ni and NiO-Ni/CeZrO_x_ catalysts.

The basic properties associated with CO_2_ adsorption capacity were investigated by CO_2_-TPD. Three kinds of basic sites were classified as weak (<150 ℃), moderate (150-450 ℃), and strong (>450 ℃) basic sites. The CeZrO_x_/NiO-Ni inverse catalyst mainly presented weak and moderate types of basic sites, corresponding to the CO_2_ adsorption on metal-oxide interface, which indicated a smaller oxide particle size.^[3]^ On the contrary, NiO-Ni/CeZrO_x_ conventional catalyst mainly presented strong adsorption at high temperature of >600℃, corresponding to the CO_2_ adsorption on bulk oxide. Generally, the moderately basic sites were closely related to the CO_2_ activation ability during the methanation process, indicating a more outstanding CO_2_ activation capability of inverse catalyst.


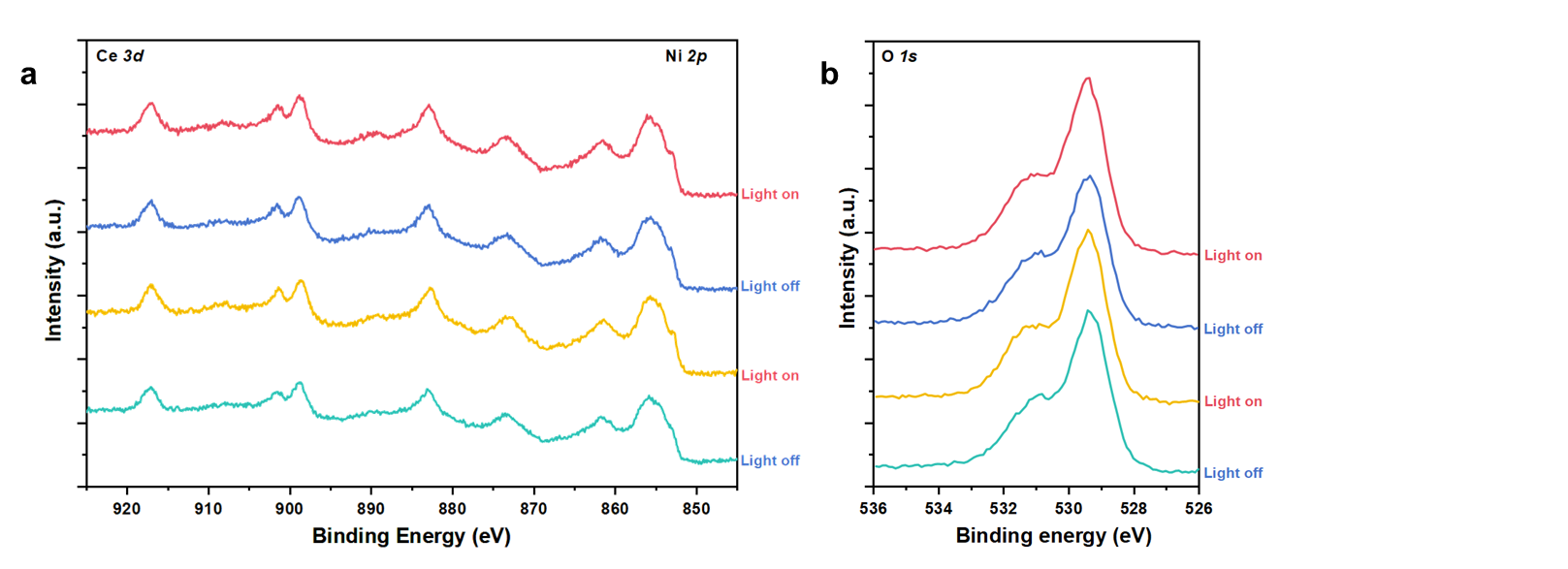


**Figure S23.** NAP-XPS results of **a.** Ce *3d* and Ni *2p*, **b.** O*1s* on CeZrO_x_/NiO-Ni catalyst under Ar atmosphere.


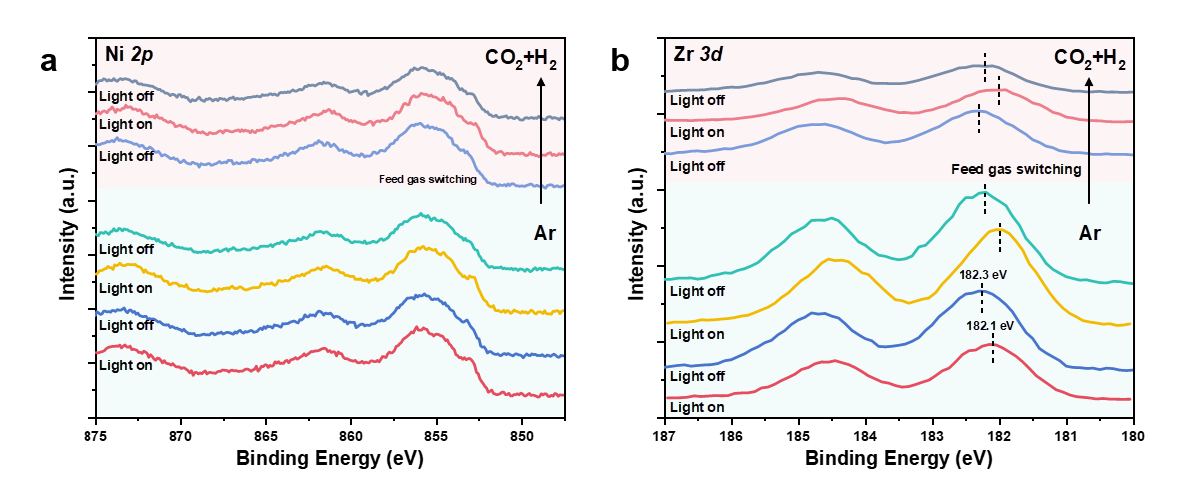


**Figure S24.** NAP-XPS results of **a.** Ni *2p* and **b.** Zr *3d* on CeZrO_x_/NiO-Ni with light off-on switching under Ar atmosphere and CO_2_+H_2_ atmosphere.

The Ni*2p* spectra obtained in the CO_2_+H_2_ atmosphere were not distinctly different from those measured in Ar, and switching the light on-off did not cause obvious change in the valence state of Ni species. The Zr *3d* peak shifted toward lower binding energy under illumination, both in Ar and CO_2_+H_2_ atmosphere, demonstrating light‑induced electron accumulation on the oxide and confirming that irradiation promotes interfacial electron transfer.


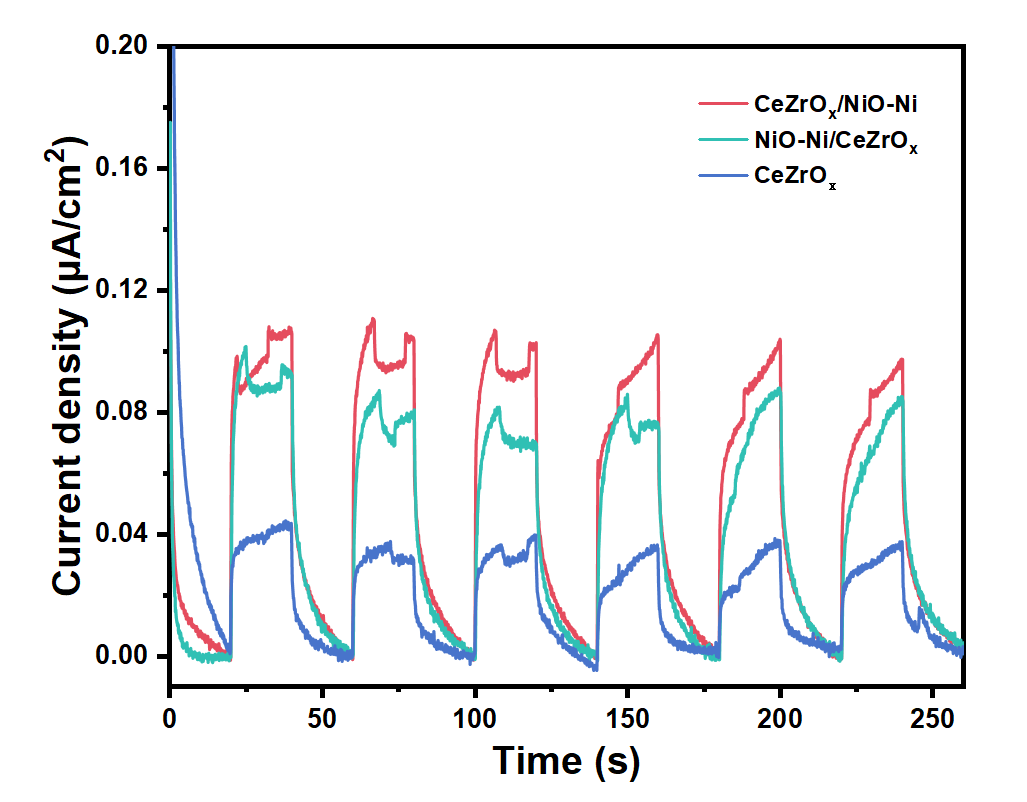


**Figure S25.** Transient photocurrent density of CeZrO_x_/NiO-Ni, NiO-Ni/CeZrO_x_ and CeZrO_x_ catalysts.

**Table S1.** The physicochemical characteristics of CeZrO_x_/NiO-Ni, NiO-Ni/CeZrO_x_, CeZrO_x_ and NiO-Ni catalysts.

| **Catalyst** | **Ni^[a]^**  **(mol%)** | **Ce^[a]^**  **(mol%)** | **Zr^[a]^**  **(mol%)** | **S_BET_^[b]^**  **(m^2^/g)** | **Total volume^[b]^**  **(cm^3^/g)** | **Average pore diameter^[b]^ (nm)** |
| --- | --- | --- | --- | --- | --- | --- |
| CeZrO_x_/NiO-Ni | 87.5 | 6.5 | 6.0 | 84.51 | 0.48 | 22.84 |
| NiO-Ni/CeZrO_x_ | 13.0 | 44.7 | 42.3 | 14.76 | 0.09 | 23.11 |
| CeZrO_x_ | - - | 51.1 | 48.9 | 16.76 | 0.16 | 38.8 |
| NiO-Ni | 100.0 | - - | - - | 42.28 | 0.27 | 25.2 |

^[a]^ Determined by ICP-AES.

^[b]^ Determined by BET method.

**Table S2.** The value of binding energy for the XPS fitting result.

| **Sample** | **Element** | | | **Binding Energy (eV)** |
| --- | --- | --- | --- | --- |
| CeZrO_x_/NiO | Ni | Ni^0^ | *2p* | -- |
|  |  | Ni^2+^ | *2p* | 853.7, 855.5, 860.9, 864.1, 866.5 |
| CeZrO_x_/Ni | Ni | Ni^0^ | *2p* | 852.4, 855.7, 861.0 |
|  |  | Ni^2+^ | *2p* | -- |
| CeZrO_x_/NiO-Ni | Ni | Ni^0^ | *2p* | 852.5, 858.5 |
|  |  | Ni^2+^ | *2p* | 853.7, 855.5, 860.9, 864.1, 866.2 |

**Supplementary References**

1. Johnson, P.B., and Christy, R.W. "Optical Constants of Transition Metals – Ti, V, Cr, Mn, Fe, Co, Ni, and Pd," *Phys. Rev. B* (1974): **12**, 5056-5070. 10.1103/PhysRevB.9.5056.
2. Franta, D., Negulescu, B., Thomas, L., et al. "Optical properties of NiO thin films prepared by pulsed laser deposition technique," *Appl. Surf. Sci.* (2005): **1**, 426-430. <https://doi.org/10.1016/j.apsusc.2004.09.150>.
3. Xu, Y., Gao, Z., Xu, Y., et al. "Cu-supported nano-ZrZnO_x_ as a highly active inverse catalyst for low temperature methanol synthesis from CO_2_ hydrogenation," *Appl. Catal. B-Environ.* (2024): 123656. <https://doi.org/10.1016/j.apcatb.2023.123656>.
4. Derry, G.N., Kern, M.E., and Worth, E.H. "Recommended values of clean metal surface work functions," *Journal of Vacuum Science & Technology A* (2015): **6**. 10.1116/1.4934685.
5. Mateo, D., Morlanes, N., Maity, P., et al. "Efficient Visible-Light Driven Photothermal Conversion of CO_2_ to Methane by Nickel Nanoparticles Supported on Barium Titanate," *Adv. Funct. Mater.* (2021): **8**, 2008244. <https://doi.org/10.1002/adfm.202008244>.
6. Ullah, S., Lovell, E., Wong, R.J., et al. "Light-Enhanced CO_2_ Reduction to CH_4_ using Nonprecious Transition-Metal Catalysts," *ACS Sustain. Chem. Eng.* (2020): **13**, 5056-5066. 10.1021/acssuschemeng.9b06823.
7. Li, P., Zhang, S., Xiao, Z., et al. "Ni-TiO_2_ catalysts derived from metal-organic framework for efficient photo-thermal CO_2_ methanation," *Fuel* (2024): 129817. 10.1016/j.fuel.2023.129817.
8. Li, Q., Gao, Y., Zhang, M., et al. "Efficient infrared-light-driven photothermal CO_2_ reduction over MOF-derived defective Ni/TiO_2_," *Appl. Catal. B-Environ.* (2022): 120905. 10.1016/j.apcatb.2021.120905.
9. Wu, Z., Li, C., Li, Z., et al. "Niobium and Titanium Carbides (MXenes) as Superior Photothermal Supports for CO_2_ Photocatalysis," *ACS Nano* (2021): **3**, 5696-5705. 10.1021/acsnano.1c00990.
10. Wang, H.L., Li, Q., Chen, J., et al. "Efficient Solar-Driven CO_2_ Methanation and Hydrogen Storage Over Nickel Catalyst Derived from Metal-Organic Frameworks with Rich Oxygen Vacancies," *Adv. Sci.* (2023): **34**, 2304406. 10.1002/advs.202304406.
11. Li, Z., Shi, R., Zhao, J., and Zhang, T. "Ni-based catalysts derived from layered-double-hydroxide nanosheets for efficient photothermal CO_2_ reduction under flow-type system," *Nano Res.* (2021): **12**, 4828-4832. 10.1007/s12274-021-3436-6.
12. Li, J., Xu, Q., Han, Y., et al. "Efficient photothermal CO_2_ methanation over NiFe alloy nanoparticles with enhanced localized surface plasmon resonance effect," *Sci. China Chem.* (2023): **12**, 3518-3524. 10.1007/s11426-023-1876-4.
13. Khan, I.S., Mateo, D., Shterk, G., et al. "An Efficient Metal–Organic Framework-Derived Nickel Catalyst for the Light Driven Methanation of CO_2_," *Angew. Chem. Int. Ed.* (2021): **51**, 26476-26482. <https://doi.org/10.1002/anie.202111854>.
14. Mateo, D., Albero, J., and García, H. "Titanium-Perovskite-Supported RuO_2_ Nanoparticles for Photocatalytic CO_2_ Methanation," *Joule* (2019): **8**, 1949-1962. <https://doi.org/10.1016/j.joule.2019.06.001>.
15. O'Brien, P.G., Sandhel, A., Wood, T.E., et al. "Photomethanation of Gaseous CO_2_ over Ru/Silicon Nanowire Catalysts with Visible and Near-Infrared Photons," *Adv. Sci.* (2014): **1**, 1400001. <https://doi.org/10.1002/advs.201400001>.
16. Ge, H., Kuwahara, Y., Kusu, K., et al. "Ru/H_x_MoO_3-y_ with plasmonic effect for boosting photothermal catalytic CO_2_ methanation," *Appl. Catal. B-Environ.* (2022): 121734. <https://doi.org/10.1016/j.apcatb.2022.121734>.
17. Ren, J., Ouyang, S., Xu, H., et al. "Targeting Activation of CO_2_ and H_2_ over Ru-Loaded Ultrathin Layered Double Hydroxides to Achieve Efficient Photothermal CO_2_ Methanation in Flow-Type System," *Adv. Energy Mater.* (2017): **5**, 1601657. 10.1002/aenm.201601657.
18. Tang, Y.X., Yang, Z.Y., Guo, C., et al. "Encapsulating Ir nanoparticles into UiO-66 for photo-thermal catalytic CO_2_ methanation under ambient pressure," *J. Mater. Chem. A* (2022): **22**, 12157-12167. 10.1039/d2ta00933a.
19. O'Brien, P.G., Ghuman, K.K., Ali, F.M., et al. "Enhanced photothermal reduction of gaseous CO_2_ over silicon photonic crystal supported ruthenium at ambient temperature," *Energy Environ. Sci.* (2018): **12**, 3443-3451. 10.1039/c8ee02347f.
20. Jiang, H., Wang, L., Kaneko, H., et al. "Light-driven CO_2_ methanation over Au-grafted Ce_0.95_Ru_0.05_O_2_ solid-solution catalysts with activities approaching the thermodynamic limit," *Nat. Catal.* (2023): **6**, 519-530. 10.1038/s41929-023-00970-z.
21. Sastre, F., Puga, A.V., Liu, L., et al. "Complete Photocatalytic Reduction of CO_2_ to Methane by H_2_ under Solar Light Irradiation," *J. Am. Chem. Soc.* (2014): **19**, 6798-6801. 10.1021/ja500924t.
